# Elasticity and stability of GdAl_2_ under pressure and temperature investigated using DFT+AI

**DOI:** 10.1038/s41598-025-99186-3

**Published:** 2025-05-04

**Authors:** Reyhaneh Ebrahimi-Jaberi, Saeid Jalali-Asadabadi

**Affiliations:** Department of Physics, Faculty of Physics, University of Isfahan (UI), Hezar Jerib Avenue, 8174673441 Isfahan, Iran

**Keywords:** Condensed-matter physics, Electronic properties and materials, Materials science, Physics, Condensed-matter physics, Electronic properties and materials

## Abstract

The cubic ferromagnetic Laves phase intermetallic compound $\text {GdAl}_2$ is a promising candidate for aerospace, defense, and advanced engineering applications due to its thermal stability and reliable elastic properties under pressure. However, two key gaps persist: discrepancies between theoretical and experimental elastic constants, and a lack of systematic pressure-dependent investigations. This study addresses these gaps, highlighting $\text {GdAl}_2$’s exceptional thermal stability, with melting temperatures rising linearly under pressure, its near-isotropic compressive behavior, and mild anisotropy in shear and Young’s moduli. Using density functional theory, elasticity theory, and AI-driven neural networks, we systematically analyzed the elasticity and stability of the system under pressure and temperature. A rigorous energy-based methodology resolves the first gap, setting a benchmark for cubic systems. To address the second gap, we analyzed mechanical stability up to 20 GPa via the Born stability criteria, finding consistent increases in elastic constants, bulk modulus, and Young’s modulus under compression. Phonon dispersion and density of states analyses confirm dynamic stability and reveal that low-frequency acoustic modes dominated by Gd atoms drive elastic behavior, reflecting spin-dominated mechanics. Poisson’s ratio shows mild anisotropy, while ductility assessments reaffirm the material’s brittle nature, consistent with Laves phase intermetallics. By integrating advanced computational methods and AI predictions, this work resolves theoretical-experimental discrepancies, establishes a framework for spin-dominated systems, and positions $\text {GdAl}_2$ as a benchmark for spin-lattice interactions and anisotropy in next-generation engineering under pressure.

## Introduction

The binary $\text {AB}_2$ family is renowned for its exceptional physical properties and versatile applications, spanning piezoelectricity^1^, thermoelectricity^2^, thermomagnetism^3^, magnetic refrigeration^4^, magnetocaloric effects^5,6^, giant magnetostriction^7^, optics^8^, magneto-optics^9^, superparamagnetism^10^, superconductivity^11^, and hydrogen storage^12^. The rare-earth-based subfamily $\text {RAl}_2$ (R = rare earth) has garnered interest for its diverse properties, including thermal^8,9^, electronic^13^, thermomagnetic^3,13^, magnetic^9,13,14^, superconductivity^9^, and the de Haas-van Alphen effect^8,9^. Notably, it exhibits large magnetocaloric entropy changes^3,15^ and is promising for environmentally friendly magnetic refrigeration^16^. $\text {GdAl}_2$, a notable member of the $\text {RAl}_2$ family, features a cubic ferromagnetic (FM) Laves phase and the highest Curie temperature ($T_C = 170$ K) among $\text {RAl}_2$ alloys^6,14,17^. It exhibits magnetically glassy and random anisotropy behaviors in nanostructured forms^18^ and serves as a model for quantum Heisenberg ferromagnetism studies^19^. $\text {GdAl}_2$ exhibits a distinct discontinuity in the thermal derivative of its longitudinal elastic constant at $T_C$, attributed to its anomalous elastic properties^14,20^, contrasting with FM materials like Invar alloys^21^. Its half-filled $4f^7$ configuration makes $\hbox {Gd}^{3+}$ a pure spin system^3^, underscoring its theoretical importance.

Although $\text {GdAl}_2$ exhibits intriguing properties, its elastic behavior, particularly under pressure, remains underexplored. Schiltz *et al.*^20^ measured its elastic constants experimentally (4-300 K), while Xiaoma *et al.*^12^ predicted them using the Perdew-Wang GGA^22^ in the VASP code^23^. However, two key gaps persist: (1) unresolved discrepancies between theoretical and experimental elastic constants, particularly $C_{11}$ and $C_{44}$, and (2) the absence of systematic studies on pressure-dependent elastic properties, crucial for assessing $\text {GdAl}_2$’s performance under pressure.

The discrepancy between theoretical and experimental elastic constants hinders the validation of models and weakens predictions for $\text {GdAl}_2$’s mechanical properties. To resolve this, we employ an energy-based methodology for calculating elastic constants^24,25^, detailed in Sec. “Mechanical stability and the critical role of spin polarization”. Traditional rhombohedral strain methods couple $C_{44}$ with the bulk modulus (*B*) via expressions like $\frac{3}{2}V_0^{-1} \frac{d^2E}{d\epsilon ^2} = C_{11} + 2C_{12} + 4C_{44} = 3B_0 + 4C_{44}$, introducing inaccuracies^24^. Our approach decouples $C_{44}$ from *B* using a single deformation, as expressed in Eq. 6 of Ref.^24^, and independent calculations (Eq. (3)), achieving precision and establishing a benchmark for elastic constant calculations in cubic systems.

To address the lack of systematic investigation into pressure-dependent elastic properties, we analyzed its mechanical response under pressures up to 15 GPa and temperatures up to 500 K. The Born stability criteria^26^ confirm that $\text {GdAl}_2$ remains mechanically stable under both compression and thermal effects, reinforcing its suitability for high-stress applications. Key parameters, including bulk modulus (*B*), Young’s modulus (*E*), shear modulus (*G*), and melting temperature ($T_m$), increase consistently with pressure, highlighting its robust performance. Poisson’s ratio reveals mild anisotropy in the (*xy*), (*xz*), and (*yz*) planes, while $\frac{B}{G}$ analysis reaffirms its brittle nature, consistent with Laves phase intermetallics^27^. Cauchy pressure and Kleinman parameter analyses indicate retained covalent bonding and resistance to lattice distortions, positioning $\text {GdAl}_2$ as a candidate for structural applications in high-stress, high-pressure environments.

To verify dynamic stability at zero pressure, we complemented mechanical stability analysis using the Born criteria^26^ with phononic studies. Phonon dispersion and density of states (DOS), computed via the harmonic force-constant method in Phonopy^28,29^, confirmed the absence of imaginary frequencies, indicating stability. The phonon DOS revealed that Gd atoms dominate low-frequency acoustic modes (0-4 THz), influencing elastic properties, while Al atoms contribute mainly to higher-frequency optical modes, reinforcing the mechanical robustness of $\text {GdAl}_2$ under zero-pressure conditions.

To complement zero-temperature DFT calculations, we employed a feedforward multi-layer perceptron neural network to model the temperature dependence of elastic constants, following our recent methodology^30^. Trained within a Bayesian regularization framework using experimental data (4-300 K)^20^, the model effectively captures nonlinear elastic behavior. Predictions almost match DFT-calculated values at zero temperature, validating our computational framework and bridging the gap between first-principles theory and experiment. This artificial intelligence (AI) driven approach yielded predictions closely matching DFT-calculated values, validating our computational framework and bridging the gap between theory and experiment. The agreement highlights the robustness of our methodology in capturing $\text {GdAl}_2$’s elastic behavior and establishes a novel framework for studying complex materials with limited experimental data, paving the way for reliable deployment in high-performance applications.

Building on prior advancements, this study explores $\text {GdAl}_2$ as a distinctive member of the $\text {AB}_2$ family, addressing limitations in our previous work^31^, including the lack of experimental validation and pressure effects. Unlike our previously studied system with pronounced anisotropy, $\text {GdAl}_2$ demonstrates near-isotropic compressive resistance and mild anisotropy in shear and Young’s moduli, making it an excellent model for investigating the relationship between structural symmetry and mechanical behavior.

By uncovering the interplay between structural symmetry, mechanical stability, and pressure-dependent behavior, this study deepens our understanding of the physical mechanisms governing elasticity in spin-dominated systems. The near-isotropic compressive response and mechanical robustness of $\text {GdAl}_2$ stem from its distinctive structural and electronic configuration, offering fundamental insights into the stability of materials under pressure. Beyond its role as a model system for exploring spin-lattice interactions and anisotropy, $\text {GdAl}_2$’s exceptional mechanical properties position it as a promising candidate for aerospace and high-performance engineering applications requiring superior stability and elasticity under challenging environments^32^.

## Theory and method

### Theoretical background of elastic calculation

Elasticity describes the reversible deformation of materials under stress, where internal restoring forces return the material to equilibrium after stress removal^33,34^. Elastic constants quantitatively capture this behavior and are critical for determining mechanical properties such as phase stability, lattice dynamics, and thermodynamic behavior^20,35–39^. These constants also govern sound velocities, entropy, and interatomic potentials, making them essential for both theoretical and practical applications in material science^3,40–42^.

Elastic constants allow the derivation of secondary properties such as the shear modulus (*G*), bulk modulus (*B*), Young’s modulus (*E*), and Poisson’s ratio ($\nu$), which are indispensable for assessing structural integrity under operational stresses^36,39–42^. These constants can be computed using two primary methods: (a) the *energy approach*, which calculates constants from variations in total energy with respect to strain^43^, and (b) the *stress-strain theorem*, which relates stress tensors ($\sigma _{ij}$) to strain tensors ($\varepsilon _{ij}$)^44^.

This study employs the energy approach, implemented in the IRelast package^25^. The package has been validated against experimental data across various symmetries and crystal structures, demonstrating its reliability^24^. For cubic systems, lattice symmetry reduces the elastic constant matrix to three independent terms: $C_{11}$, $C_{12}$, and $C_{44}$^25^. These constants describe the material’s mechanical response and are derived by fitting the total energy of a strained system to a Taylor expansion based on Hooke’s law. The elastic constants are determined from the second derivatives of energy with respect to strain as:

1 $$\begin{aligned} \begin{aligned}&\frac{\partial ^2 E}{\partial \varepsilon ^2} = 2 V_0 (C_{11} - C_{12}), \end{aligned} \end{aligned}$$

2 $$\begin{aligned} \begin{aligned}&\frac{\partial ^2 E}{\partial \varepsilon ^2} = 3 V_0 (C_{11} + 2 C_{12}), \end{aligned} \end{aligned}$$

3 $$\begin{aligned} \begin{aligned}&\frac{\partial ^2 E}{\partial \varepsilon ^2} = 4 V_0 C_{44}. \end{aligned} \end{aligned}$$

The IRelast package uses predefined deformation matrices to compute $C_{11}$, $C_{12}$, and $C_{44}$ for cubic crystals^25^. The calculations are performed using the full-potential augmented plane waves plus local orbitals (FP-APW+lo) method, implemented in the WIEN2k package^45–47^. This methodology ensures high accuracy and consistency with experimental results, providing a robust framework for analyzing the elastic properties of cubic systems under various conditions.

### Neural networks method

**Fig. 1:**
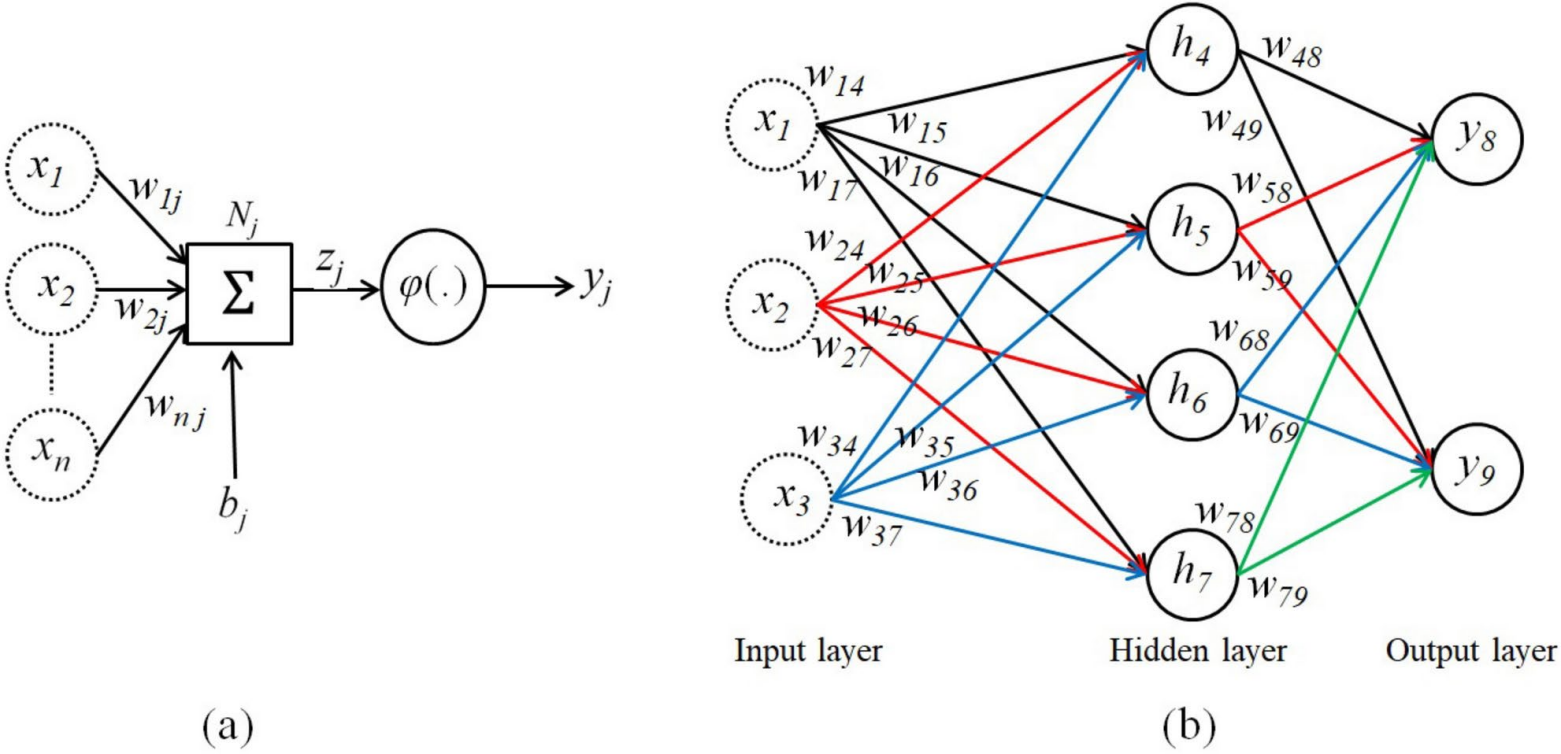
(**a**) Schematic model of a single artificial neuron in a neural network. The neuron receives multiple inputs ($x_1, x_2,..., x_n$) weighted by $w_{ij}$, which represent the strength of the connection between input $x_i$ and neuron $N_j$. The weighted inputs are summed, and a bias term $b_j$ is added to produce a total input $z_j$ to the neuron. This input is then passed through an activation function $\varphi (.)$, generating the output $y_j$, which is propagated through the network. (**b**) A schematic representation of a fully connected feedforward neural network. While the diagram illustrates a simple multi-layer perceptron (MLP) with a single hidden layer for clarity, our implementation employs a deeper architecture with two hidden layers, each consisting of 16 neurons, and utilizes rectified linear unit (ReLU) activation functions. The network is trained using backpropagation within a Bayesian Regularization framework, which optimizes a regularized loss function to balance accuracy and generalization. The structure depicted here demonstrates the fundamental principles of MLP architectures in solving complex problems by transforming input data into a desired output through a series of weighted transformations and nonlinear activations.

Neural networks are advanced AI tools that model complex nonlinear relationships, first conceptualized by McCulloch and Pitts in the 1940s^48^. These models consist of interconnected neurons that process inputs ($x_i$) via weighted connections ($w_{ij}$) to produce outputs ($y_j$), as shown in Fig. 1(a). Each neuron applies an activation function $\varphi (z_j)$ to the weighted input sum ($z_j$), which includes a bias term ($b_j$)^49^:

4 $$\begin{aligned} \begin{aligned} y_j = \varphi (z_j) = \varphi \left( \sum _{i=0}^n x_i w_{ij}\right) . \end{aligned} \end{aligned}$$

The use of activation functions such as the rectified linear unit (ReLU) introduces nonlinearity, enabling neural networks to capture intricate behaviors^49^. Fig. 1(b) provides a schematic representation of a feedforward multi-layer perceptron (MLP) with input, hidden, and output layers. While the figure illustrates a simplified architecture with a single hidden layer, our implementation employs two hidden layers with 16 neurons each, utilizing ReLU activations to model the temperature dependence of elastic constants^30^. Neural networks are trained using backpropagation, which minimizes the error between predictions (${\hat{y}}_j^k$) and targets ($y_j^k$). This study follows our Bayesian Regularization framework, which penalizes large weights to prevent overfitting and enhance generalization. The total loss function is defined as:$\text {Total Loss} = E_D + \lambda E_W,$ where $E_D$ represents the mean squared error (MSE) between predicted and experimental values, and $E_W$ is a regularization term that penalizes large weights. By optimizing this loss function, the model effectively balances accuracy and generalization, ensuring reliable predictions across a wide range of conditions.

By neural networks, we predict elastic properties with acceptable accuracy. This method bridges experimental data from 4-300 K with zero-temperature predictions^49^.

### Details of calculations

**Fig. 2:**
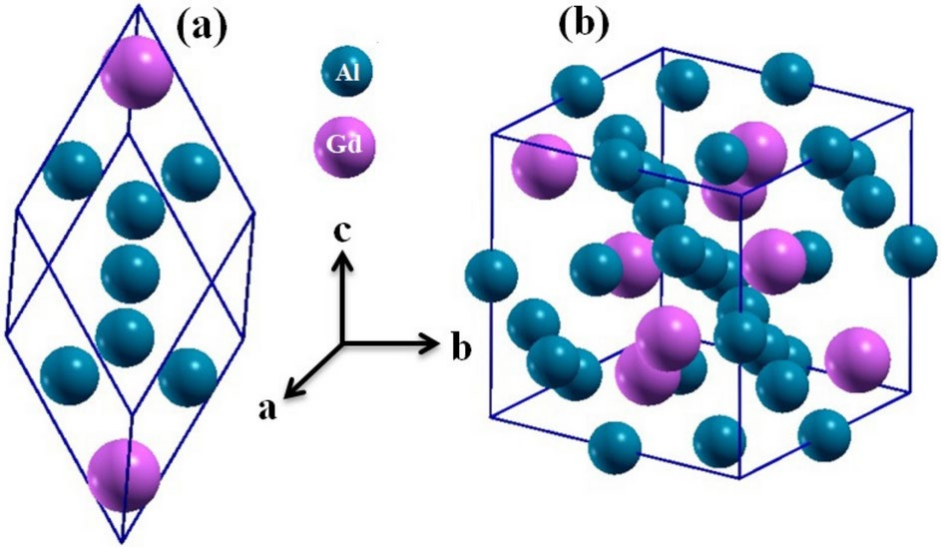
(**a**) Primitive and (**b**) conventional unit cells of the cubic Laves phase of $\text {GdAl}_2$.

We employed density functional theory (DFT) within the WIEN2k package^46,47^ to investigate the elastic and electronic properties of $\text {GdAl}_2$, which crystallizes in the cubic $\text {MgCu}_2$ (C15) Laves phase (space group Fd${\bar{3}}$m), as shown in Fig. 2^50,51^. Our study focuses on its ferromagnetic (FM) phase, which is stable below the Curie temperature of approximately 170 K^51^.

Calculations utilized the FP-APW+lo method^45^, solving the Kohn-Sham equations^52,53^ with exchange-correlation functionals including LDA, PBE-GGA, PBEsol-GGA, and WC-GGA^22,54–57^. To address the localized 4f orbitals of Gd, we incorporated Hubbard *U* corrections ($U_{\text {eff}}$) ranging from 1 to 9 eV^58,59^.

The lattice parameters were optimized using the Murnaghan equation of state^60^, and the elastic constants ($C_{11}$, $C_{12}$, $C_{44}$) were computed using the IRelast package^25^, extending the cubic-elastic code^24^. Calculations were based on a plane-wave cutoff of $K_{\text {max}} = 9.5/R_{\text {MT}}$, muffin-tin radii of $R_{\text {MT}} = 2.3~\text {bohr}$, and a $16 \times 16 \times 16$ Monkhorst-Pack grid^61^. These elastic constants were used to derive mechanical properties such as the bulk modulus (*B*), shear modulus (*G*), Young’s modulus (*E*), and Poisson’s ratio ($\nu$)^24,25^.

Neural networks^62,63^ were employed to predict experimental elastic constants at zero temperature, leveraging elastic data from 4-300 K. The networks were trained using a Bayesian Regularization-enhanced backpropagation algorithm^64^, reducing overfitting and ensuring accurate predictions.

Dynamical stability was assessed using the Phonopy package^28,29^, employing a $2 \times 2 \times 2$ supercell to calculate phonon dispersion and DOS. This analysis confirmed $\text {GdAl}_2$’s stability and revealed atom-specific contributions to its elastic properties.

## Analysis of elastic constants of GdAl_2_ and their extensive consequences

This section presents a detailed exploration of the elastic and mechanical properties of the cubic Laves phase of $\hbox {GdAl}_2$, guiding through a multi-faceted analysis of this fascinating material. The discussion begins with an investigation into its mechanical stability, where we uncover the critical influence of spin polarization on accurately modeling the material’s elastic properties. Through this, we demonstrate that, while spin-orbit coupling and the effective Hubbard parameter are often considered, they exert minimal impact compared to spin polarization. This sets the stage for a deep understanding of how spin effects govern the mechanical behavior of $\hbox {GdAl}_2$. Recognizing the limitations of standard density functional theory at different temperatures, we take the analysis further by integrating neural networks. This innovative approach allows us to predict elastic constants over a range of temperatures, pushing beyond the static capabilities of DFT to achieve results that align more closely with experimental data. Here, the synergy between machine learning and first-principles methods shows how modern computational techniques can bridge the gap between theory and experiment.

The discussion then shifts to the interplay between phonon dynamics and elasticity, where we delve into the phonon dispersion and density of states to explore the material’s dynamical stability. This section reveals the atomistic contributions, particularly from Gd atoms, that shape the elastic behavior, offering deep insights into the fundamental interactions within the material’s lattice. From this, we transition into a mechanical evaluation of $\hbox {GdAl}_2$, extracting key properties such as bulk modulus, shear modulus, and Vicker’s hardness. These derived properties not only quantify the material’s resistance to deformation but also provide a comprehensive picture of its mechanical integrity, offering insights into its potential applications in various industries. The section also explores the sound velocities, melting temperature, and Debye temperature, uncovering how these thermal and acoustic properties are intertwined with the material’s elastic constants. This analysis illuminates the thermal robustness and vibrational characteristics of $\hbox {GdAl}_2$, crucial for understanding its behavior in real-world applications.

A discussion on elastic anisotropy follows, where we assess the directional dependence of $\hbox {GdAl}_2$’s elastic properties. Interestingly, the material exhibits near isotropic behavior in compression, with only minimal anisotropy in shear, suggesting that it can perform reliably under various mechanical stresses. Finally, we close the section with an examination of how pressure affects $\hbox {GdAl}_2$’s elastic properties. The material remains mechanically stable under pressures up to 15 GPa, and we observe notable increases in elastic constants, phonon velocities, and melting temperature. These findings not only confirm the material’s robustness but also extend our understanding of its performance under pressure.

### Mechanical stability and the critical role of spin polarization

This section investigates the elastic properties of $\hbox {GdAl}_2$ using a range of XC functionals, including LDA, PBE-, PBEsol-, and WC-GGA, with a particular focus on SP, SOC, and $U_{\text {eff}}$, see Table 1. The symbols in this table correspond to the following notes: [$^\pitchfork$ Scalar relativistic all-electron Bl$\ddot{\text {o}}$chl’s projector augmented-wave approach, $^\maltese$ Perdew-Wang parameterization (PW91) version of GGA, implemented in the Vienna *ab initio* simulation package (VASP), $^\Supset$ Measured at 4 K, $^\blacklozenge$ Taken from Refs.^65^ and^66^, and $^\clubsuit$ See Sec. 3.9, where the temperature dependence of elastic constants is discussed.] The elastic constants, namely $C_{11}$, $C_{12}$, and $C_{44}$, are crucial for assessing the mechanical stability and structural integrity of cubic crystals like $\hbox {GdAl}_2$. According to the Born stability criteria for cubic systems^26^, the following conditions must be satisfied to ensure mechanical stability:

5 $$\begin{aligned} \begin{aligned} C_{11} - C_{12}> 0, \quad C_{11} + 2C_{12}> 0, \quad C_{44} > 0. \end{aligned} \end{aligned}$$

By applying these criteria, considering the results tabulated in Table 1, we confirm that $\hbox {GdAl}_2$ is mechanically stable at zero temperature and pressure under all considered computational methods.

**Table 1:** Elastic constants of $\text {GdAl}_2$ calculated using various exchange-correlation functionals (LDA, PBEsol-, WC-, and PBE-GGA+SP+U(+SOC)) for $U_{\text {eff}} = 1.00$ to 9.00 eV, with comparisons to available theoretical and experimental data. Here, SP, SOC, and $U_{\text {eff}}$ denote spin-polarization, spin-orbit coupling, and the effective Hubbard parameter, respectively. Data in parentheses reflect SOC-inclusive calculations. Results from this study are marked by *, with optimal values, XC functionals, corresponding experimental data at 4K, and neural network results at zero temperature. To streamline the table, blank cells within each block indicate values identical to the preceding cell, excluding superscripted data and irrelevant cells tied to experimental results.

Scheme	Method	XC	SP	SOC	$U_{\text {eff}}~(\text {eV})$	$C_{11}~(\text {GPa})$	$C_{12}~(\text {GPa})$	$C_{44}~(\text {GPa})$	*a* (Å)	Ref.
DFT	APW+lo	LDA	No	No		166.38	36.86	25.61	7.628	*
		PBEsol				157.81	33.37	35.24	7.716	*
		WC				155.97	33.72	33.59	7.723	*
		PBE				138.39	35.96	34.58	7.816	*
		LDA	Yes	No (Yes)		179.85 (180.63)	38.56 (38.98)	65.08 (65.03)	7.751 (7.747)	*
		PBEsol				173.56 (173.18)	35.55 (35.76)	63.74 (64.07)	7.830 (7.826)	*
		WC				170.53 (171.25)	35.78 (35.94)	62.79 (62.86)	7.837 (7.833)	*
		PBE				161.31 (161.55)	33.23 (32.94)	58.90 (59.13)	7.914 (7.910)	*
		LDA+U			1	181.68 (182.69)	38.94 (39.44)	66.13 (65.67)	7.754 (7.749)	*
					2	181.90 (183.56)	38.72 (39.71)	65.77 (66.14)	7.758 (7.753)	*
					3	182.61 (183.76)	39.16 (39.75)	66.22 (66.32)	7.761 (7.757)	*
					4	182.59 (183.66)	39.17 (39.78)	66.14 (66.05)	7.765 (7.761)	*
					5	182.43 (183.53)	39.15 (39.74)	65.96 (65.92)	7.769 (7.765)	*
					6	182.29 (183.31)	39.12 (39.71)	65.67 (65.87)	7.772 (7.768)	*
					7	182.11 (183.19)	39.09 (39.67)	65.55 (67.64)	7.775 (7.770)	*
					8	181.92 (183.02)	39.05 (39.68)	65.41 (65.59)	7.779 (7.773)	*
					9	181.72 (182.83)	39.06 (39.62)	65.23 (45.49)	7.780 (7.776)	*
		PBEsol+U			1	173.95 (174.43)	35.98 (36.17)	64.67 (64.33)	7.833 (7.829)	*
					2	174.39 (174.96)	36.15 (36.32)	64.41 (64.33)	7.837 (7.833)	*
					3	174.44 (174.95)	36.18 (36.38)	64.25 (64.35)	7.840 (7.837)	*
					4	174.39 (174.87)	36.18 (36.31)	64.14 (64.21)	7.844 (7.840)	*
					5	174.15 (175.10)	36.15 (36.08)	63.76 (64.08)	7.848 (7.844)	*
					6	174.03 (174.51)	36.12 (36.28)	63.55 (63.92)	7.851 (7.847)	*
					7	173.84 (174.35)	36.10 (36.21)	63.41 (63.69)	7.853 (7.850)	*
					8	173.69 (174.16)	36.07 (36.19)	63.29 (63.70)	7.856 (7.853)	*
					9	173.55 (174.04)	36.03 (36.19)	63.19 (63.48)	7.859 (7.855)	*
		WC+U			1	172.00 (172.47)	36.21 (36.40)	63.50 (63.18)	7.839 (7.836)	*
					2	172.52 (173.03)	36.42 (36.56)	63.47 (63.54)	7.843 (7.840)	*
					3	172.53 (173.07)	36.47 (36.61)	63.11 (63.46)	7.847 (7.843)	*
					4	172.43 (172.97)	36.43 (36.60)	62.99 (63.19)	7.850 (7.847)	*
					5	172.20 (172.73)	36.41 (36.59)	62.77 (63.08)	7.854 (7.851)	*
					6	172.09 (172.57)	36.37 (36.54)	62.68 (62.85)	7.857 (7.854)	*
					7	171.92 (172.43)	36.39 (36.51)	62.53 (62.76)	7.860 (7.857)	*
					8	171.73 (173.44)	36.36 (36.86)	62.44 (63.10)	7.863 (7.859)	*
					9	171.60 (172.05)	36.31 (36.43)	62.31 (62.44)	7.865 (7.862)	*
	$\hbox {PAW}^\pitchfork$	$\hbox {GGA}^\maltese$		No		163.07	35.38	51.15	7.917	^12^
		Neural network$^\clubsuit$				169.44	34.07	64.29		*
Exp.$^\Supset$						169.26	34.92	64.61	7.900$^\blacklozenge$	^20^

The introduction of $U_{\text {eff}}$, which accounts for the on-site Coulomb interaction within Gd’s localized 4f orbitals, has a nuanced impact on the elastic constants rather than resulting in a simple, consistent increase, see Table 1. Initially, as $U_{\text {eff}}$ increases (e.g., from 0 to 1 eV), we observe a modest rise in the elastic constants, such as an increase in $C_{11}$ from 170.53 GPa to 172.00 GPa using WC-GGA+SP+*U*. However, further increases in $U_{\text {eff}}$ lead to diminishing returns, with values stabilizing or even slightly decreasing at higher $U_{\text {eff}}$ values (e.g., at $U_{\text {eff}} = 9 \, \text {eV}$, $C_{11} = 171.60 \, \text {GPa}$, $C_{12} = 36.31 \, \text {GPa}$, and $C_{44} = 62.31 \, \text {GPa}$). Interestingly, even without applying $U_{\text {eff}}$, the computed elastic constants, particularly with the WC-GGA+SP functional, align closely with experimental data. For example, the calculated values of $C_{11} = 170.53 \, \text {GPa}$, $C_{12} = 35.78 \, \text {GPa}$, and $C_{44} = 62.79 \, \text {GPa}$ with WC-GGA+SP (without $U_{\text {eff}}$) are in agreement with experimental measurements (i.e., $C_{11} = 169.26 \, \text {GPa}$, $C_{12} = 34.92 \, \text {GPa}$, and $C_{44} = 64.61 \, \text {GPa}$). This suggests that additional $U_{\text {eff}}$ adjustments may not significantly improve accuracy for this compound. Thus, the WC-GGA+SP functional alone captures the balance between electronic localization and mechanical stability without the need for applying $U_{\text {eff}}$, providing results that closely approximate experimental values. Therefore, in the case of GdAl$_2$, using WC-GGA+SP without $U_{\text {eff}}$ can be a reliable approach to model its elastic properties. However, in contrast to $U_{\text {eff}}$, SP is essential for accurately modeling the elastic properties of $\hbox {GdAl}_2$, which is expected given the magnetic nature of Gd and the importance of spin in its electronic structure. Thus, the inclusion of SP is necessary to capture the correct mechanical response of $\hbox {GdAl}_2$.

The impact of SOC on the elastic properties is relatively small, see Table 1. For example, when SOC is included in WC-GGA+SP calculations (without $U_{\text {eff}}$), the predicted elastic constants change only slightly: $C_{11} = 171.25 \, \text {GPa}$, $C_{12} = 35.94 \, \text {GPa}$, and $C_{44} = 62.86 \, \text {GPa}$. These values are only marginally different from the non-SOC values of $C_{11} = 170.53 \, \text {GPa}$, $C_{12} = 35.78 \, \text {GPa}$, and $C_{44} = 62.79 \, \text {GPa}$. The minor changes indicate that SOC plays a negligible role in the elastic behavior of $\hbox {GdAl}_2$, likely due to the fact that $\hbox {Gd}^{3+}$’s 4f electrons are localized and the SOC effects are not dominant in this context^67^.

The experimental lattice constant for $\text {GdAl}_2$ is $a = 7.900 \, \text {{\text{\AA }}}$^20^. In our study, we initially observed that multiple functionals, including PBE-, PBEsol-, and WC-GGA, provided lattice constants close to this experimental value, see Table 1. For example, PBE-GGA predicts $a = 7.914 \, \text {{\text{\AA }}}$ (without SOC) and $a = 7.910 \, \text {{\text{\AA }}}$ (with SOC), while WC-GGA+SP (without SOC) predicts $a = 7.837 \, \text {{\text{\AA }}}$. Incorporating the effective Hubbard parameter ($U_{\text {eff}}$) with several functionals further improved the agreement with the experimental lattice constant, highlighting the versatility of DFT in tuning lattice properties. To ensure a rigorous assessment, we extended our comparison by calculating elastic constants ($C_{11}$, $C_{12}$, and $C_{44}$) for all functionals that yielded reasonable lattice parameters. This broader approach allowed us to assess which functional could best capture both the lattice constant and the mechanical properties, as the latter are more sensitive indicators of a material’s bonding and structural response. Our findings showed that WC-GGA+SP provided results closest to the experimental values for both the lattice constant and the elastic constants, particularly $C_{11}$ and $C_{44}$, which are challenging to predict accurately. This consistency indicates that WC-GGA+SP not only approximates the atomic spacing accurately but also captures the underlying bonding interactions responsible for the material’s mechanical stability. Other functionals, although yielding lattice parameters close to experiment, exhibited larger deviations in elastic constants, making WC-GGA+SP the most reliable choice when considering both properties together. Moreover, WC-GGA+SP maintains this accuracy without requiring adjustments to parameters such as $U_{\text {eff}}$ or SOC. While some functionals with SOC or specific $U_{\text {eff}}$ values provided similar lattice constants, these modifications often led to less accurate elastic constants or less consistency across properties. WC-GGA+SP’s performance with minimal parameter tuning demonstrates an intrinsic compatibility with the structural and electronic environment of $\text {GdAl}_2$. To further evaluate WC-GGA+SP’s robustness, we analyze pressure-dependent behavior for bulk modulus (*B*), shear modulus (*G*), and Young’s modulus (*E*). WC-GGA+SP consistently provided results that aligned well with theoretical expectations and showed realistic trends under increasing pressure, indicating its capability to accurately describe $\text {GdAl}_2$’s response in both ambient and high-pressure environments. Therefore, WC-GGA+SP is selected as the preferred functional due to its balanced performance across lattice parameters, elastic constants, and pressure-dependent mechanical properties. This reliability across a broad range of properties underscores WC-GGA+SP’s suitability for studying $\text {GdAl}_2$ comprehensively. Thus, although various functionals offer lattice constants close to experimental values, WC-GGA+SP stands out for its superior balance, accurately predicting both lattice and elastic properties without needing additional tuning. This makes WC-GGA+SP the optimal functional for capturing $\text {GdAl}_2$’s mechanical behavior comprehensively, supporting its application in environments where both high stiffness and structural stability are crucial.

In summary, based on the results presented in Table 1, the WC-GGA+SP functional offers the most accurate predictions for the elastic properties of $\hbox {GdAl}_2$, with calculated values $C_{11} = 170.53 \, \text {GPa}$, $C_{12} = 35.78 \, \text {GPa}$, and $C_{44} = 62.79 \, \text {GPa}$ closely matching the experimental values $C_{11} = 169.26 \, \text {GPa}$, $C_{12} = 34.92 \, \text {GPa}$, and $C_{44} = 64.61 \, \text {GPa}$. While including SOC and $U_{\text {eff}}$ slightly refines the results, their overall impact is minimal. These findings suggest that the WC-GGA+SP functional is the most suitable for capturing both the elastic constants and the lattice constant of $\hbox {GdAl}_2$ with high accuracy, outperforming other functionals such as LDA, PBE-, and PBEsol-GGA. The close agreement with experimental data underscores the reliability of this computational approach in describing the mechanical behavior of $\hbox {GdAl}_2$.

### Integrating neural networks with DFT to enable temperature-dependent predictions of elastic properties

While density functional theory (DFT) is a highly reliable tool for calculating electronic structure and elastic properties, its standard formulation inherently operates at zero temperature, lacking the capability to account for temperature-dependent variations. To address this limitation, we incorporated neural networks into our methodology, as detailed in Secs. 2.2 and 3.9. This integration bridges the gap between static DFT predictions and experimental data, enabling dynamic, temperature-sensitive predictions that reflect real-world conditions. Specifically, the neural networks were trained on experimental data in the 4-300 K range^20^, allowing for the estimation of elastic constants at various temperatures with high accuracy.

The neural network architecture employed in this study, illustrated in Fig. 1(b), was designed to complement the WIEN2k and IRelast DFT calculations. The input was a $9 \times 1$ matrix of temperature values, while the output was a $9 \times 3$ matrix of elastic constants. A feedforward neural network with one hidden layer containing 10 neurons was utilized, and the backpropagation algorithm iteratively adjusted the weights of the connections to minimize prediction error. To prevent overfitting and ensure generalization to unseen data, the Bayesian Regularization algorithm was employed^64^. This robust design enabled the neural network to accurately capture the relationship between temperature and elastic constants.

Table 1 demonstrates the effectiveness of this approach, showing that the neural network predictions for elastic constants at zero temperature were in agreement with experimental data measured at 4 K. For example, the experimental values of $C_{11} = 169.26$ GPa, $C_{12} = 34.92$ GPa, and $C_{44} = 64.61$ GPa were almost replicated by the model. This level of agreement underscores the reliability of the neural network in complementing DFT calculations and compensating for their zero-temperature limitation. Beyond simple temperature extrapolation, the integration of neural networks offers several advantages that enhance the overall accuracy and efficiency of the methodology: (*i*) The neural networks refine DFT predictions by aligning them with experimental observations, addressing minor discrepancies often associated with exchange-correlation functional approximations. (2) By using experimental data, the neural networks provide a dynamic, temperature-sensitive model of $\hbox {GdAl}_2$’s mechanical properties without requiring computationally expensive ab initio molecular dynamics simulations. (*iii*) The neural networks capture nonlinear relationships between temperature and elastic properties, enabling precise predictions over a wide range of conditions.

This synergy between machine learning and DFT is particularly evident in the WC-GGA functional, which demonstrated the best agreement with experimental results (Table 1). Neural network integration further enhanced this functional’s predictive accuracy, offering a comprehensive model of $\hbox {GdAl}_2$’s behavior across varying temperatures and pressures. By enabling temperature-dependent predictions and aligning them with experimental data, as discussed in Sec. 3.9, the integration of neural networks pushes the boundaries of computational materials science. This innovative combination bridges the gap between theoretical and experimental domains, offering a versatile framework for studying materials under real-world conditions. The integration highlights how artificial intelligence complements traditional computational techniques, allowing for dynamic and high-accuracy predictions of temperature-sensitive properties. This approach not only addresses the limitations of standard DFT calculations but also opens new avenues for applying machine learning in materials design.

### Phonon-elastic interplay for mechanical and dynamical stability through phonon dispersion and DOS at zero pressure

**Fig. 3:**
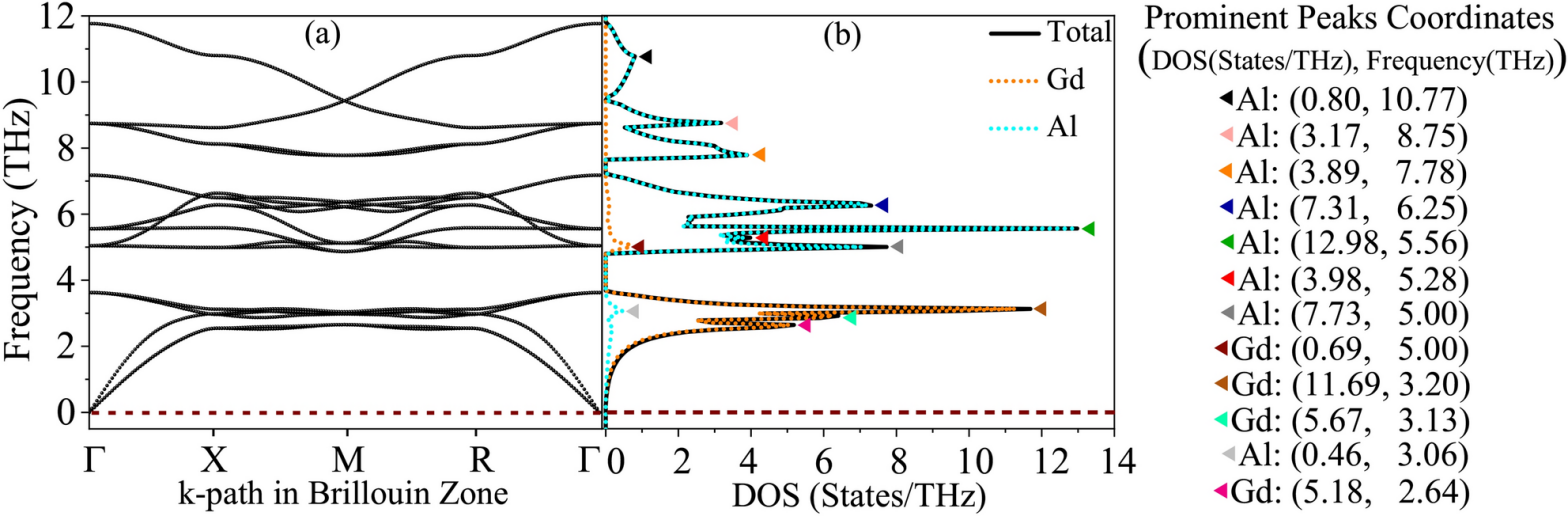
(**a**) Phonon dispersion of $\text {GdAl}_2$, illustrating the variation of phonon frequency with wave vector across different directions in the Brillouin zone. (**b**) Atom-resolved phonon density of states (DOS) for $\text {GdAl}_2$, highlighting the contributions of Gd and Al atoms to the phonon spectrum. The prominent peaks and their coordinates in the DOS-frequency space are explicitly marked in the figure legend. These features provide insights into the vibrational characteristics and atomistic contributions to the lattice dynamics. Calculations were performed at zero pressure using the WC-GGA+SP method, where SP denotes spin polarization.

The elastic constants of a material are closely linked to its phonon dispersion and density of states (DOS). The phonon dispersion and DOS were computed at zero pressure using the WC-GGA+SP functional, as shown in Figs. 3(a) and (b). Mahdi Sanati and coauthors^36^ have systematically studied elastic constants, phonon density of states, and thermal properties of UO$_2$ in the DFT framework using LDA+U and GGA+U, as implemented in the pseudopotential-based VASP code^23,68^. M. Sanati *et al.* pointed out that the elastic constants are proportional to the slopes of the linear low-frequency acoustic phonon modes, which play a significant role in determining the mechanical behavior of the system. For a compound like $\text {GdAl}_2$ with six atoms per unit cell, the number of phonon modes is given by 3*N*, where *N* is the number of atoms, resulting in three acoustic modes and fifteen optical modes^69^.

The vibrational dynamics of $\text {GdAl}_2$, depicted in Fig. 3, provide critical insights into its mechanical and thermal properties. The phonon dispersion in Fig. 3(a) spans a frequency range of 0-12 THz, with a clear distinction between the low-frequency acoustic modes (0-4 THz) and the high-frequency optical modes (7-12 THz). The acoustic modes, originating at the $\Gamma$ point, represent long-wavelength vibrations where adjacent atoms move coherently. These modes are integral to the elastic properties, reflecting the material’s stiffness and resistance to deformation under mechanical stress. The slopes of the acoustic branches are relatively steep, suggesting strong interatomic forces and significant contributions to the elastic constants. Additionally, the absence of imaginary phonon frequencies throughout the Brillouin zone confirms the dynamical stability of $\text {GdAl}_2$, ensuring its robustness under perturbations. The atom-resolved phonon density of states (DOS), shown in Fig. 3(b), highlights the distinct contributions of Gd and Al atoms across the frequency spectrum. This detailed breakdown provides insights into the material’s vibrational characteristics, which are directly linked to its elastic properties, thermal transport, and potential applications in advanced materials design.

In the low-frequency acoustic region, [0, 4 THz], the dominance of Gd atoms is apparent due to their larger mass. The most prominent Gd-related peak at 3.20 THz, with a DOS value of 11.69 States/THz, underscores their critical role in supporting acoustic modes and low-energy optical vibrations. These modes are key contributors to the material’s stiffness and thermal transport at low frequencies. The small Al-related peak at 3.06 THz, with a value of 0.46 States/THz, indicates a minor contribution from Al in this range. The negligible role of Al in the acoustic region aligns with its lighter atomic mass, which limits its vibrational contributions at lower frequencies. The intermediate frequency range, [4, 7 THz], marks a transition between acoustic and optical modes. Here, a noticeable shift in dominance occurs as Al atoms play a more prominent role. The most significant peak in this region, located at 5.56 THz with a value of 12.98 States/THz, highlights Al’s increasing influence. Meanwhile, Gd-related contributions diminish, as seen in the smaller peak at 5.00 THz with a value of 0.69 States/THz. This transition zone reflects a complex interplay of both atomic species, with potential implications for mixed-mode thermal transport. The influence of Al atoms in this range is particularly important for understanding vibrational modes that mediate between low-frequency elastic behavior and high-frequency thermal dynamics. In the high-frequency optical region, [7, 12 THz], the dominance of Al atoms becomes pronounced. Due to their lighter mass, Al atoms govern high-energy optical vibrations, with a notable peak at 7.78 THz (3.89 States/THz). Beyond 7 THz, the lattice dynamics are almost exclusively controlled by Al atoms, as seen by the clustering of Al-related peaks at higher frequencies. These modes are essential for understanding the material’s high-frequency vibrational characteristics, which can influence thermal conductivity and phonon scattering processes. Gd’s contributions in this range are negligible, reflecting its inertial limitations at high frequencies. Optimizing Al’s vibrational dynamics in this regime could enhance $\text {GdAl}_2$’s performance in applications requiring high-frequency vibrational properties, such as thermal energy management.

The combined analysis of phonon dispersion and atom-resolved DOS reveals the distinct vibrational roles of Gd and Al in $\text {GdAl}_2$, offering a roadmap for tailoring its mechanical and thermal properties. By manipulating the relative concentrations of Gd and Al, the material’s acoustic and optical vibrational characteristics could be optimized for specific applications. For example, enhancing Gd’s contributions could improve the material’s performance in low-energy acoustic wave propagation or thermal transport, while leveraging Al’s dominant high-frequency modes could optimize the material for high-energy phonon scattering or advanced thermal management systems. The absence of imaginary frequencies, combined with the material’s robust elastic constants and steep acoustic branch slopes, confirms its suitability for applications requiring mechanical resilience and stability under pressure. These findings position $\text {GdAl}_2$ as a promising candidate for advanced engineering applications, including thermal management, acoustic devices, and high-pressure or high-temperature environments where material stability is critical.

### Mechanical insights and hardness evaluation from elastic constants to Vicker’s hardness and beyond

The elastic constants provide key insights into the mechanical properties of solids, offering a pathway to calculate various critical parameters that characterize material behavior under stress. One such parameter is the Cauchy pressure ($P_{c}$), which for cubic crystals is defined as^72^:

6 $$\begin{aligned} \textit{P}_{c} = C_{12} - C_{44}. \end{aligned}$$

Pettifor demonstrated that the sign of the Cauchy pressure can indicate the nature of bonding in a material: positive values typically correspond to metallic bonding, while negative values suggest covalent bonding^73^. Our results, as shown in Table 2, reveal negative Cauchy pressure values for $\text {GdAl}_2$ using WC-GGA+SP, indicating the presence of covalent bonds between atoms in this compound. The symbols in this table correspond to the following notes: [$^\Cap$ The Perdew-Wang parameterization (PW91) version of GGA, as implemented in the Vienna ab initio Simulation Package (VASP). $^\maltese$ The atomic-sphere approximation (ASA) using an spdf basis. $^\bigstar$ The open-core treatment. $^\circledast$ Miedema’s semiempirical model, also known as the macroscopic atom model. $\dagger$ The Von Barth-Hedin (VBH) version of LDA, applied using the augmented spherical wave (ASW) method. $^\lozenge$ The full-potential linear muffin-tin orbital (LMTO) method with gradient correction. $^\boxtimes$ The Langreth-Mehl-Hu gradient correction added to LDA.]

**Table 2:** The Cauchy pressure ($P_{c}$), bulk modulus ($\text {B}$), shear modulus ($\text {G}$), ductility ratio ($\text {B/G}$), Young’s modulus ($\text {E}$), Poisson’s ratio ($\nu$), Kleinman’s internal displacement parameter ($\zeta$), Vicker’s hardness ($\text {H}_{V}$), melting temperature ($T_{m}$), and Debye temperature ($\Theta _{D}$) of $\text {GdAl}_2$, calculated using WC-GGA+SP, are provided. Results from this study are marked with *. To avoid redundancy, blank cells within each block represent repeated values from the cell immediately above, except for superscripted values and cells related to experimental data. SP and SOC indicate spin-polarization and spin-orbit coupling, respectively.

Scheme	Method	XC	SP	SOC	$P_{c}~(\text {GPa})$	$\text {B}~(\text {GPa})$	$\text {G}~(\text {GPa})$	$\text {B/G}$	$\text {E}~(\text {GPa})$	$\nu$	$\zeta$	$\text {H}_{V}~(\text {GPa})$	$T_{m}$ ± 300 (K)	$\Theta _{D}$ (K)	Ref.
DFT	APW+lo	WC	Yes	No	-27.00	80.70	64.58	1.25	152.94	0.18	0.36	23.57	1560.83	401.98	*
	PAW	$\hbox {GGA}^\Cap$				77.94	55.90		135.35	0.21				376.90	^12^
	TB-LMTO	$\hbox {LDA}^\maltese$				86.50									^17^
						83.0$^\bigstar$									^17^
	ASW	LDA$\dagger$				86.00									^70^
	$\hbox {FLG}^\lozenge$	$\hbox {LDA}^\boxtimes$				76.00									^70^
Emp.$^\circledast$						78.00$^\circledast$									^71^
Exp.						75.30									^14^
						80.00									^70^
										0.19				406.00	^20^

Two additional parameters, bulk modulus (*B*) and shear modulus (*G*), are closely related to elastic constants and offer further insight into the mechanical stability of materials. Bulk modulus (*B*) measures the resistance of a material to volume changes under uniform pressure, while shear modulus (*G*) quantifies its resistance to shape deformation under shear stress^38^. These moduli can be calculated using the Voigt-Reuss-Hill approximation^74–76^, which provides an average estimate based on the assumptions of uniform strain (Voigt) and uniform stress (Reuss) across the polycrystal^77,78^.

For cubic crystals, the Voigt bulk modulus ($B_{V}$) and shear modulus ($G_{V}$) are defined as follows^77^:

7 $$\begin{aligned} & \textit{B}_\textit{V} = \frac{1}{3} \left( C_{11} + 2~C_{12}\right) , \end{aligned}$$

8 $$\begin{aligned} & \textit{G}_\textit{V} = \frac{1}{5} \left( C_{11} - C_{12} + 3~C_{44}\right) . \end{aligned}$$

Similarly, the Reuss bulk modulus ($B_{R}$) and shear modulus ($G_{R}$) are given by^77^:

9 $$\begin{aligned} & \textit{B}_\textit{R} = \frac{1}{3~S_{11} + 6~S_{12}}, \end{aligned}$$

10 $$\begin{aligned} & \textit{G}_\textit{R} = \frac{15}{4~S_{11} - 4S_{12} + 3S_{44}}, \end{aligned}$$

where $S_{ij}$ are the elastic compliance constants for cubic crystals, which can be expressed as^79^:

11 $$\begin{aligned} & \textit{S}_\textit{11} = \frac{C_{11} + C_{12}}{\left( C_{11} - C_{12}\right) \left( C_{11} + 2~C_{12}\right) }, \end{aligned}$$

12 $$\begin{aligned} & \textit{S}_\textit{12} = \frac{-C_{12}}{\left( C_{11} - C_{12}\right) \left( C_{11} + 2~C_{12}\right) }, \end{aligned}$$

13 $$\begin{aligned} & \textit{S}_\textit{44} = \frac{1}{C_{44}}. \end{aligned}$$

Hill’s approximation suggests that the effective bulk and shear moduli should be the arithmetic averages of the Voigt and Reuss estimates, as given by^76^:

14 $$\begin{aligned} & B = \frac{1}{2} \left( B_{V} + B_{R}\right) , \end{aligned}$$

15 $$\begin{aligned} & G = \frac{1}{2} \left( G_{V} + G_{R}\right) . \end{aligned}$$

Our calculated values of *B* using WC-GGA+SP, presented in Table 2, are in good agreement with experimental data. Since the bulk modulus is larger than the shear modulus, it indicates that $\text {GdAl}_2$ has stronger resistance to volume change than to shape deformation.

The ductility of a material can also be assessed through the ratio of bulk modulus to shear modulus (*B*/*G*), as proposed by Pugh^80^. If $B/G > 1.75$, the material is considered ductile; otherwise, it is brittle^78^. Our calculated *B*/*G* values, shown in Table 2, reveal that $\text {GdAl}_2$ is brittle, which is consistent with previous studies on Laves phase materials^27^.

Young’s modulus (*E*) and Poisson’s ratio ($\nu$) are additional mechanical properties derived from the bulk and shear moduli. Young’s modulus measures the stiffness of a material, while Poisson’s ratio quantifies the material’s stability against shear deformation. These are defined as^81–83^:

16 $$\begin{aligned} & E = \frac{9BG}{3B+G}, \end{aligned}$$

17 $$\begin{aligned} & {\nu } = \frac{3B-2G}{6B+2G}. \end{aligned}$$

The Poisson’s ratio ($\nu$) of a material, typically ranging between -1 and 0.5, is a key indicator of its stability against shear deformation. In this range, a higher Poisson’s ratio suggests greater plasticity, whereas lower values point to brittleness. Generally, values of $\nu$ close to 0.5 indicate highly ductile materials, while values approaching 0 indicate less ductility. According to Frantsevich’s rule^84^, materials with $\nu > \frac{1}{3}$ are considered ductile, and those with $\nu < \frac{1}{3}$ are classified as brittle. For $\text {GdAl}_2$, the Poisson’s ratio is less than $\frac{1}{3}$, as shown in Table 2, indicating its brittle nature. This result is consistent with the *B*/*G* ratio for $\text {GdAl}_2$, another metric that confirms brittleness in materials. Therefore, both $\nu$ and *B*/*G* ratios classify $\text {GdAl}_2$ as brittle, and this assessment aligns well with experimental values for Poisson’s ratio, which further supports the brittle classification for $\text {GdAl}_2$.

Kleinman’s internal displacement parameter ($\zeta$), which indicates how rigidly atoms maintain their relative positions under strain, is defined as^85^:

18 $$\begin{aligned} {\zeta } = \frac{C_{11} + 8C_{12}}{7C_{11} + 2~C_{12}}. \end{aligned}$$

The Kleinman internal displacement parameter ($\zeta$) offers insight into the flexibility of atomic positions under volume-conserving strain distortions. Physically, $\zeta$ measures the relative rigidity of atomic bonds within the lattice: $\zeta = 0$ implies fixed atomic positions during distortions, while nonzero values indicate that atoms can adjust their positions internally under strain. For $\text {GdAl}_2$, our calculated $\zeta$ values using WC-GGA+SP, shown in Table 2, deviate from zero, suggesting that atomic positions are not entirely rigid and can undergo displacement in response to lattice distortions. In practical terms, a nonzero $\zeta$ value indicates that bond lengths and angles may adjust when $\text {GdAl}_2$ is subjected to stress, reflecting a degree of atomic movement. However, the values of $\zeta$ remain significantly below unity, indicating limited flexibility. This limited internal displacement aligns with $\text {GdAl}_2$’s classification as a brittle material, as seen from the previously discussed Poisson’s ratio ($\nu$) and *B*/*G* ratio. The deviation from $\zeta = 1$ for bond length invariance and $\zeta = -\frac{1}{2}$ for bond angle invariance^72^ further confirms that $\text {GdAl}_2$ does not accommodate large-scale deformations easily. Instead, the atomic structure exhibits only slight positional adjustments, underscoring a material rigidity that resists significant rearrangement under strain. This rigidity is consistent with a brittle response to applied stress, as bond-breaking is more likely than bond-flexing when the lattice is deformed. In other words, the nonzero $\zeta$ value for $\text {GdAl}_2$ suggests moderate atomic flexibility under stress, but the low magnitude of $\zeta$ aligns with its brittle nature. The calculated $\zeta$ thus provides additional confirmation that $\text {GdAl}_2$ lacks the atomic-level adaptability typically associated with ductile materials, reinforcing its classification as brittle based on its mechanical response.

We also calculate the Vicker’s hardness parameter ($H_{V}$), a key indicator of a material’s resistance to plastic deformation, using the following expression^86^:

19 $$\begin{aligned} \textit{H}_\textit{V} = 0.92 ~ {\left( \frac{B}{G}\right) }^{~1.3137} G^{~0.708}. \end{aligned}$$

This formula accounts for both the bulk modulus (*B*) and shear modulus (*G*), allowing $H_{V}$ to capture the interplay between volume resistance and shape deformation resistance. Typically, *B* reflects a material’s resistance to uniform compression, while *G* indicates resistance to shear or deformation under non-uniform stress. Together, these moduli influence hardness, as $H_{V}$ incorporates the material’s overall structural rigidity. A material is classified as superhard if $H_{V}$ exceeds 40 GPa^87^. According to Table 2, the calculated $H_{V}$ values for $\text {GdAl}_2$ are significantly below this threshold, confirming that $\text {GdAl}_2$ is not a superhard material. This hardness result indicates that, while $\text {GdAl}_2$ may have moderate resistance to wear and indentation, it lacks the extreme hardness needed for applications that require superhard materials, such as cutting tools or abrasives. However, $\text {GdAl}_2$’s moderate $H_{V}$ does suggest it could be suitable for applications where moderate hardness is advantageous, especially in conditions where wear resistance and thermal stability are critical. For instance, its hardness, combined with its thermal stability, may make $\text {GdAl}_2$ a strong candidate for protective coatings or components that experience consistent surface contact under moderate stress. Nevertheless, the brittleness of $\text {GdAl}_2$—as indicated by both its $H_{V}$ and *B*/*G* ratios—implies that it may not perform well in applications requiring significant ductility or tolerance to deformation under high loads. The low Poisson’s ratio and brittle classification mean that $\text {GdAl}_2$ is likely to fracture under heavy stress, limiting its use in load-bearing or impact-prone structures. Therefore, while $\text {GdAl}_2$ does not achieve the hardness level required to classify it as superhard, its moderate hardness and thermal stability may make it well-suited for specialized applications where wear resistance is beneficial, provided that brittleness is not a limiting factor.

### Sound velocities, melting and Debye temperature

Sound velocities in a cubic crystal can be derived from the elastic constants and are calculated along different crystallographic directions. These velocities provide important insights into the material’s response to mechanical waves, where the specific sound velocities in the [100], [110], and [111] directions can be expressed as follows^88^:

**Table 3:** Phonon velocities (*V*) in various directions, including transverse elastic wave velocity ($V_{T}$), longitudinal elastic wave velocity ($V_{L}$), and average wave velocity ($V_{ave}$) for $\text {GdAl}_2$, calculated using WC-GGA+SP, are presented in units of (m/s). Results from this study are marked with *. To avoid redundancy, blank cells within each table block indicate repeated values from the cell directly above. SP and SOC denote spin polarization and spin-orbit coupling, respectively.

Scheme	Method	SP	SOC	*V* (m/s)	$\hbox {GGA}^\Cap$	WC-GGA	Ref.
DFT	APW+lo	Yes	No	$V_{L}^{\left[ 100\right] }$		5408.41	*
				$V_{T}^{\left[ 100\right] }$		3281.74	*
				$V_L^{\left[ 110\right] }$		5335.21	*
				$V_{T1}^{\left[ 110\right] }$		3281.74	*
				$V_{T2}^{\left[ 110\right] }$		4807.55	*
				$V_{L}^{\left[ 111\right] }$		5310.59	*
				$V_{T}^{\left[ 111\right] }$		3360.67	*
				$V_{T}$		3328.31	*
				$V_{L}$		5349.08	*
				$V_{ave}$		3668.23	*
	PAW			$V_{ave}$	3474.70		^12^

For the [100] direction:

20 $$\begin{aligned} & V_{T1} ^{\left[ 100\right] } = V_{T2}^{ \left[ 100\right] } \equiv V_{T}^{ \left[ 100\right] } = \sqrt{\frac{C_{44}}{\rho }}, \end{aligned}$$

21 $$\begin{aligned} & V_{L} ^{\left[ 100\right] } = \sqrt{\frac{C_{11}}{\rho }}, \end{aligned}$$

where $V_{T}$ and $V_{L}$ denote the transverse and longitudinal wave velocities, respectively, and $\rho$ is the density of the material.

For the [110] direction:

22 $$\begin{aligned} V_{T1} ^{\left[ 110\right] } = \sqrt{\frac{C_{44}}{\rho }}, \end{aligned}$$

23 $$\begin{aligned} V_{T2} ^{\left[ 110\right] } = \sqrt{\frac{C_{11} - C_{12}}{\rho }}, \end{aligned}$$

24 $$\begin{aligned} V_{L} ^{\left[ 110\right] } = \sqrt{\frac{C_{11} + C_{12} + 2C_{44}}{\rho }}. \end{aligned}$$

For the [111] direction:

25 $$\begin{aligned} V_{T1} ^{\left[ 111\right] } = V_{T2} ^{\left[ 111\right] } \equiv V_{T} ^{\left[ 111\right] } = \sqrt{\frac{C_{11} - C_{12} + C_{44}}{3\rho }}, \end{aligned}$$

26 $$\begin{aligned} V_{L} ^{\left[ 111\right] } = \sqrt{\frac{C_{11} + 2C_{12} + 4C_{44}}{3\rho }}. \end{aligned}$$

Using a calculated density $\rho$ of approximately $5829.7550 \, \text {kg/m}^3$ for $\text {GdAl}_2$, these equations allow us to compute the sound velocities in the respective crystallographic directions. The longitudinal sound velocity $V_L$, which represents compressional motion along the wave direction, is consistently higher than the transverse sound velocities $V_T$, which involve shear motion perpendicular to the wave direction. This difference is due to the inherent propagation mechanisms, where longitudinal waves typically encounter less resistance and thus propagate faster than shear waves. Table 3 provides our calculated values of $V_L$ and $V_T$ for $\text {GdAl}_2$ across these crystallographic directions, confirming the expected trend of $V_L > V_T$. These calculated sound velocities not only highlight the material’s structural characteristics but also have implications for its acoustic and elastic behavior, which are critical for applications involving high-frequency mechanical waves and vibrational stability. The symbol in Table 3 corresponds to the following note: [$^\Cap$ The Perdew-Wang parameterization (PW91) version of GGA, as implemented in the Vienna ab initio Simulation Package (VASP).]

The average wave velocity ($V_{ave}$) in a material is a key parameter in understanding its acoustic properties and is calculated as^89^:

27 $$\begin{aligned} V_{ave} = \left[ \frac{1}{3} \left( \frac{1}{V_{L}^3} + \frac{2}{V_{T}^3} \right) \right] ^{-\frac{1}{3}}, \end{aligned}$$

where $V_{L}$ and $V_{T}$ represent the longitudinal and transverse sound velocities, respectively, and are given by

28 $$\begin{aligned} & V_{L} = \left( \frac{3B + 4G}{3\rho } \right) ^{\frac{1}{2}}, \end{aligned}$$

29 $$\begin{aligned} & V_{T} = \left( \frac{G}{\rho } \right) ^{\frac{1}{2}}, \end{aligned}$$

where $\rho$ is the density of the material.

**Table 4:** The calculated anisotropic index of $\text {GdAl}_2$ using WC-GGA+SP. To avoid redundancy, blank cells in each table block indicate repeated values from the corresponding cell directly above. SP and SOC represent spin polarization and spin-orbit coupling, respectively.

Scheme	Method	SP	SOC	Quantity	WC-GGA
DFT	APW+lo	Yes	No	$\hbox {A}^U$	0.006
				$A_{B}$	0.000
				$A_{G}$	0.001
				$A_{1}$	0.932
				$A_{2}$	0.932
				$A_{3}$	0.932

**Table 5:** The minimum and maximum values of linear compressibility ($\beta$), Young’s modulus (*E*), shear modulus (*G*), and Poisson’s ratio ($\nu$) for $\text {GdAl}_2$ calculated using WC-GGA+SP. “Anisotropy” denotes the ratio of maximum to minimum values of $\beta$, *E*, *G*, and $\nu$. “1st Axis” indicates the primary coordinate direction (x, y, or z) where the maximum and minimum values for each parameter are observed, while “2nd Axis” represents the secondary coordinate for *G* and $\nu$. Data presented here are derived from Figs. 4 and 5. The abbreviation SP refers to spin polarization.

Scheme	XCF	SP	SOC		Linear compressibility$((\text {TPa})^{-1})$		Young’s modulus$(\text {GPa})$		Shear modulus$(\text {GPa})$		Poisson’s ratio	
					$\beta _{\text {min}}$	$\beta _{\text {max}}$	$E_{\text {min}}$	$E_{\text {max}}$	$G_{\text {min}}$	$G_{\text {max}}$	$\nu _{\text {min}}$	$\nu _{\text {max}}$
DFT	WC-GGA	Yes	No	Value	4.131	4.131	149.570	158.110	62.786	67.370	0.166	0.207
				Anisotropy	$\beta _{\text {max}}/\beta _{\text {min}}=1.000$		$E_{\text {max}}/E_{\text {min}}=1.057$		$G_{\text {max}}/G_{\text {min}}=1.073$		$\nu _{\text {max}}/\nu _{\text {min}}=1.247$	
				1st Axis	$\text {x}=0.7071$	$\text {x}=0.0000$	$\text {x}=-0.5774$	$\text {x}=0.0000$	$\text {x}=-0.0000$	$\text {x}=0.7071$	$\text {x}=0.7071$	$\text {x}=0.7071$
					$\text {y}=0.0000$	$\text {y}=0.0000$	$\text {y}=0.5774$	$\text {y}=0.0000$	$\text {y}=-0.0000$	$\text {y}=0.0001$	$\text {y}=-0.0001$	$\text {y}=-0.0000$
					$\text {z}=0.7071$	$\text {z}=1.0000$	$\text {z}=0.5773$	$\text {z}=1.0000$	$\text {z}=1.0000$	$\text {z}=-0.7071$	$\text {z}=0.7071$	$\text {z}=-0.7071$
				2nd Axis					$\text {x}=1.0000$	$\text {x}=-0.7071$	$\text {x}=0.0001$	$\text {x}=-0.7071$
									$\text {y}=0.0002$	$\text {y}=-0.0002$	$\text {y}=1.0000$	$\text {y}=0.0002$
									$\text {z}=0.0000$	$\text {z}=-0.7071$	$\text {z}=0.0000$	$\text {z}=-0.7071$

The average wave velocity $V_{ave}$ is essential for calculating the Debye temperature, thermal conductivity, and elastic stability of the material. Physically, $V_{L}$ and $V_{T}$ represent the speeds at which sound waves propagate through the material under different modes of vibration. Longitudinal waves ($V_{L}$) involve compressional motion, where atoms move along the direction of wave propagation, while transverse waves ($V_{T}$) involve shear motion, where atoms move perpendicular to the direction of propagation. Our calculations for $V_{T}$, $V_{L}$, and $V_{ave}$, using WC-GGA with spin polarization, are presented in Table 3. Consistent with typical acoustic behavior in crystalline solids, the results reveal that in $\text {GdAl}_2$, the longitudinal sound velocity $V_{L}$ is higher than the transverse sound velocity $V_{T}$. We also deduce that the fastest transverse wave and longitudinal wave are along the [110] and [100] crystal directions, respectively. This directional anisotropy highlights the dependence of acoustic wave propagation on the crystalline orientation, a feature that could have implications in material design and applications requiring directional acoustic properties. This difference between $V_{L}$ and $V_{T}$ is expected, as longitudinal waves encounter less resistance when compressing and expanding the lattice structure compared to the shearing motion of transverse waves, resulting in faster propagation for longitudinal waves. The calculated $V_{ave}$ provides an average velocity metric that is significant for several material properties. A higher average wave velocity often correlates with a higher Debye temperature, which in turn indicates enhanced thermal stability and stiffness of the material. Consequently, our results for $V_{ave}$, shown in Table 3, support the assessment of $\text {GdAl}_{2}$ as a structurally robust material with favorable acoustic properties. The higher $V_{L}$ relative to $V_{T}$ also underscores the anisotropic propagation of sound waves in different directions, a factor that could be important in applications requiring precise acoustic and thermal management. Together, these wave velocities offer a comprehensive view of the elastic and acoustic characteristics of $\text {GdAl}_2$, underscoring its stability and potential applicability in environments where thermal and acoustic properties are critical.

**Fig. 4:**
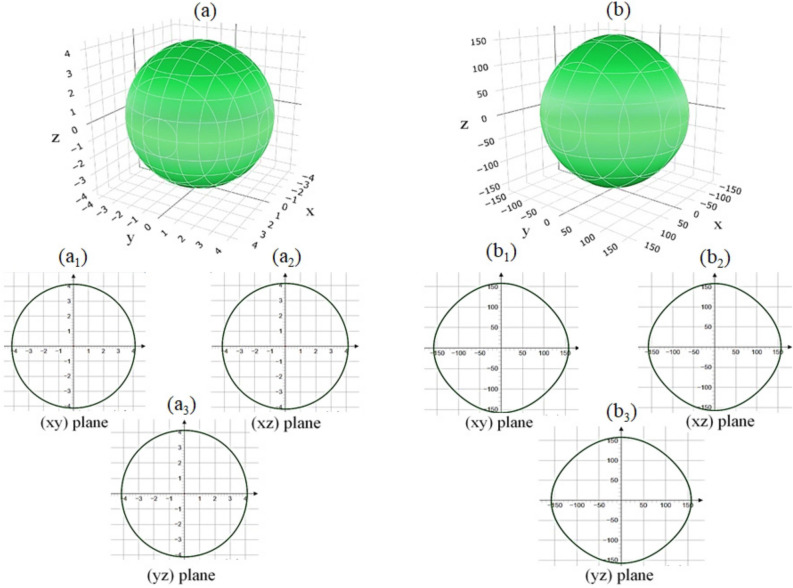
Spatial dependence of (**a**) linear compressibility (calculated using Eq. (39)) and (**b**) Young’s modulus (calculated using Eq. (40)) along the ($\text {a}_{1}$, $\text {b}_{1}$) (*xy*), ($\text {a}_{2}$, $\text {b}_{2}$) (*xz*), and ($\text {a}_{3}$, $\text {b}{3}$) (*yz*) planes for $\text {GdAl}_2$, determined using the WC-GGA+SP method. SP denotes spin polarization.

The melting temperature $T_m$ of $\text {GdAl}_2$ can be estimated from its elastic constant $C_{11}$ using an empirical relationship developed by Fine *et al.*^90^. This relationship connects $T_m$ (in Kelvin) with $C_{11}$ (in GPa) as follows:

30 $$\begin{aligned} T_m = 553 + 5.91 C_{11}, \end{aligned}$$

where $C_{11}$ represents the elastic stiffness of the crystal along one principal axis. This relationship leverages the observation that stiffer materials, represented by higher $C_{11}$ values, generally exhibit higher melting temperatures.

In our study, the elastic constant $C_{11}$ for $\text {GdAl}_2$ was calculated as 170.53 GPa using the WC-GGA+SP method. Substituting this value into Eq. 30, we obtain, see Table 2:

31 $$\begin{aligned} T_m = 553 + 5.91 \times 170.53 = 1560.83 \, \text {K}. \end{aligned}$$

This empirical estimate has an associated range of validity of $\pm 300 \, \text {K}$, meaning that the melting temperature $T_m$ is predicted to lie between 1258.83 K and 1858.83 K. Although no experimental data for $T_m$ of $\text {GdAl}_2$ currently exists, this calculation provides a preliminary reference for future investigations. The predicted melting temperature falls within the expected range for intermetallic compounds, which supports the potential of $\text {GdAl}_2$ for high-temperature applications where thermal stability is essential. In essence, the calculated $T_m$ offers insight into $\text {GdAl}_2$’s resilience under high temperatures, reinforcing its viability for applications requiring structural integrity and thermal stability at elevated temperatures.

The Debye temperature ($\Theta _D$) is a fundamental thermodynamic property that provides insights into a material’s lattice vibrations and thermal behavior. We calculate ($\Theta _D$) using the average phonon velocity ($V_{ave}$), as given by^89^:

32 $$\begin{aligned} \Theta _{D} = \frac{\hbar }{k_B} \left( \frac{3 n N_A \rho }{4 \pi M} \right) ^{1/3} V_{ave}. \end{aligned}$$

where *n* is the number of atoms per formula unit, $N_A$ is Avogadro’s number, $\rho$ is the density, and *M* is the molecular weight of $\text {GdAl}_2$.

Physically, the Debye temperature is linked to the maximum phonon frequency and, by extension, to the thermal conductivity, specific heat, and lattice stability of a material. A higher $\Theta _D$ generally correlates with stronger atomic bonds, lower atomic mobility, and higher melting points, all of which contribute to the thermal and mechanical stability of a material at elevated temperatures. For $\text {GdAl}_2$, the calculated value of $\Theta _D = 401.98 \, \text {K}$, as reported in Table 2, is in close agreement with the experimental value of $406.00 \, \text {K}$ from Ref.^20^. This good alignment with experimental data suggests that the calculated value accurately reflects the inherent phonon characteristics and bond strengths within the $\text {GdAl}_2$ crystal structure. The high Debye temperature of $\text {GdAl}_2$ indicates its suitability for high-temperature applications. Materials with elevated $\Theta _D$ values generally exhibit enhanced resistance to thermal shock and are less susceptible to thermal degradation, as their atomic vibrations are well-contained within a stable lattice. This stability makes $\text {GdAl}_2$ an attractive candidate for environments where both thermal endurance and structural integrity are critical, such as in high-temperature coatings, electronic components, or structural materials for aerospace and automotive applications. Furthermore, $\Theta _D$ serves as a benchmark for assessing a material’s thermal conductivity. Materials with high $\Theta _D$ often display high lattice thermal conductivities, which are advantageous in applications requiring efficient heat dissipation. Thus, the calculated and experimental values of $\Theta _D$ for $\text {GdAl}_2$ provide further evidence of its potential for applications where consistent thermal and mechanical performance is essential, underscoring its suitability for use in demanding thermal environments.

### Quantifying elastic anisotropy and mechanical behavior using WC-GGA+SP

Elastic anisotropy, derived from elastic constants, plays a vital role in predicting material performance under mechanical stress. It directly impacts the formation of microcracks in crystals, making it a key property for engineering applications requiring structural integrity^91^. To quantify anisotropy, Ranganathan and Ostoja-Starzewski^92^ proposed the universal anisotropy factor, $A^U$, defined as:

33 $$\begin{aligned} \begin{aligned} A^U = 5 \frac{G_V}{G_R} + \frac{B_V}{B_R} - 6. \end{aligned} \end{aligned}$$

Here, $G_V$ and $G_R$ are the Voigt and Reuss shear moduli, while $B_V$ and $B_R$ are the bulk moduli. A value of $A^U = 0$ indicates perfect isotropy, with deviations reflecting the degree of anisotropy. For $\text {GdAl}_2$, our WC-GGA+SP calculations yield $A^U = 0.006$, signifying near-isotropic behavior, as shown in Table 4. Such low anisotropy ensures uniform mechanical properties, crucial for load-bearing applications in aerospace and automotive industries.

Specific measures of anisotropy for bulk compression ($A_B$) and shear deformation ($A_G$) provide additional insights:

34 $$\begin{aligned} \begin{aligned} A_B = \frac{B_V - B_R}{B_V + B_R}, \quad A_G = \frac{G_V - G_R}{G_V + G_R}. \end{aligned} \end{aligned}$$

For $\text {GdAl}_2$, $A_B = 0$, confirming isotropy under compressive stress, while $A_G = 0.001$ reflects slight shear anisotropy, as summarized in Table 4. This minor deviation suggests that while $\text {GdAl}_2$ resists shear deformation uniformly across most directions, there is a slight directional dependence due to varying atomic bonding strengths across crystallographic planes.

Further quantification of anisotropy in specific planes ({100}, {010}, {001}) is achieved using directional shear anisotropy factors ($A_1$, $A_2$, $A_3$)^35^:

35 $$\begin{aligned} \begin{aligned} A_1 = \frac{4C_{44}}{C_{22} + C_{33} - 2C_{13}}, \quad A_2 = \frac{4C_{55}}{C_{22} + C_{11} - 2C_{23}}, \quad A_3 = \frac{4C_{66}}{C_{11} + C_{22} - 2C_{12}}. \end{aligned} \end{aligned}$$

**Fig. 5:**
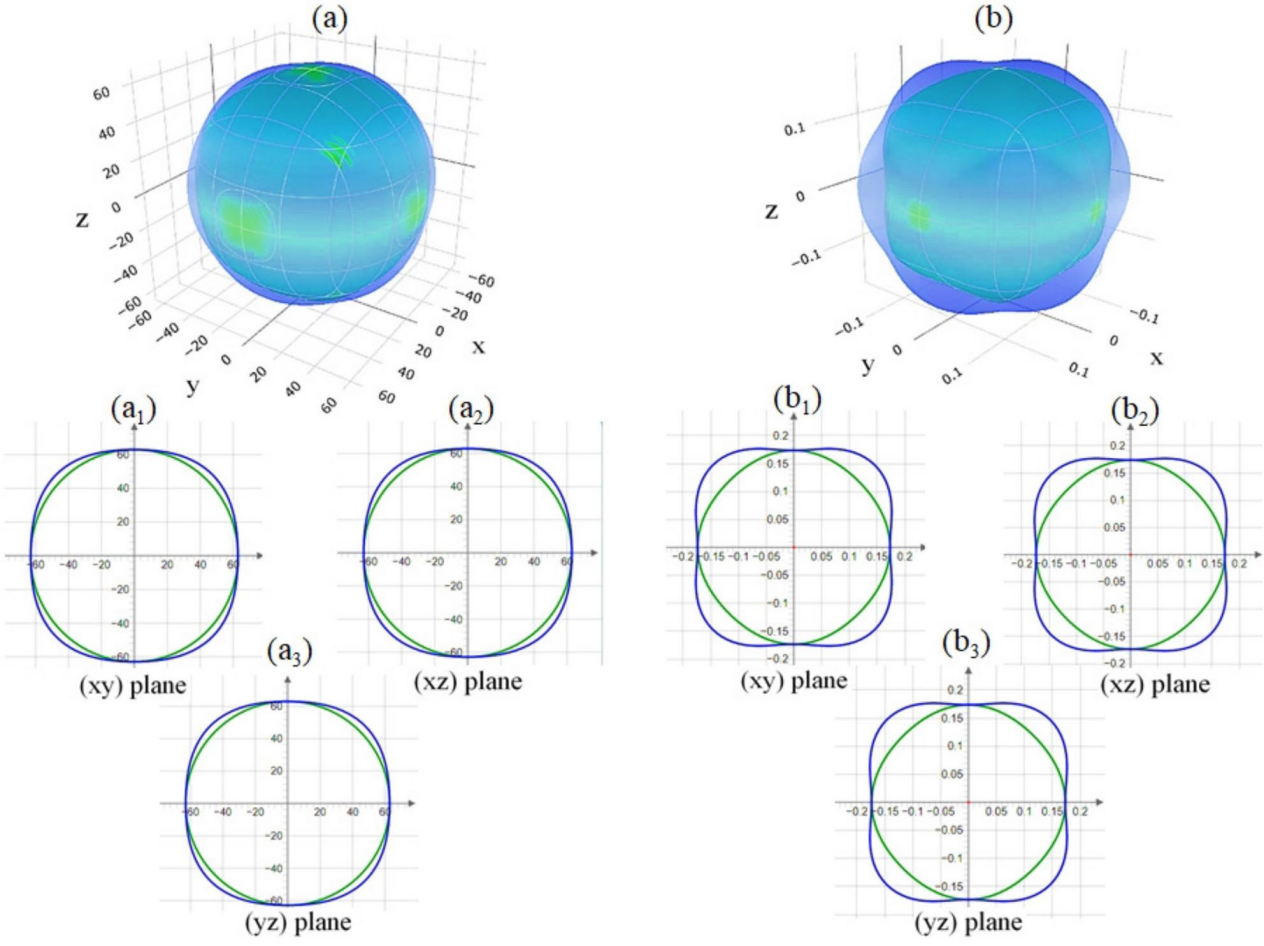
Spatial dependence of (**a**) shear modulus (calculated using Eq. (41)) and (**b**) Poisson’s ratio (calculated using Eq. (42)) along the ($\text {a}_{1}$, $\text {b}_{1}$) (*xy*), ($\text {a}_{2}$, $\text {b}_{2}$) (*xz*), and ($\text {a}_{3}$, $\text {b}_{3}$) (*yz*) planes for $\text {GdAl}_2$, determined using the WC-GGA+SP method. SP denotes spin polarization.

For an isotropic material, these values equal 1. For $\text {GdAl}_2$, our results, $A_1 = A_2 = A_3 = 0.932$, indicate slight anisotropy in bonding along specific planes, see Table 4. Although these deviations suggest weaker bonding in certain directions, the material remains nearly isotropic, making it suitable for applications requiring consistent mechanical behavior across multiple axes. The spatial dependence of the shear modulus and Poisson’s ratio, as illustrated in Fig.5, further validates the near-isotropic behavior, which is discussed in detail in Sec. “Spatial dependence of elastic properties through compressibility, moduli, and Poisson’s ratio using WC-GGA+SP”. Despite small variations across different planes, $\text {GdAl}_2$ demonstrates mechanical stability under diverse stress conditions.

In summary, $\text {GdAl}_2$ exhibits near-isotropic behavior, as evidenced by the low universal anisotropy factor ($A^U = 0.006$) and minimal deviations in directional indices ($A_B$, $A_G$, $A_1$, $A_2$, $A_3$). These results highlight its suitability for high-performance applications requiring uniform mechanical properties, such as in aerospace, automotive, and electronics industries.

### Spatial dependence of elastic properties through compressibility, moduli, and Poisson’s ratio using WC-GGA+SP

Here, we study the spatial dependence of linear compressibility ($\beta$), Young’s modulus (*E*), shear modulus (*G*), and Poisson’s ratio ($\nu$) along the (*xy*), (*xz*), and (*yz*) planes of the $\text {GdAl}_2$ crystal using the ELATE code^93^. Before diving into the spatial analysis of these elastic parameters, let us briefly discuss the theoretical foundation governing their spatial dependence.

In elastic calculations, the compliance transformation for any material from the reference system ($S_{ijkl}$) to the distorted system ($S'_{\alpha \beta \gamma \delta }$) can be expressed as follows^94^:

36 $$\begin{aligned} \begin{aligned} S'_{\alpha \beta \gamma \delta } = a_{\alpha i} a_{\beta j} b_{\gamma k} b_{\delta l} S_{ijkl}, \end{aligned} \end{aligned}$$

where, the coordinates of the two vectors ${\textbf {a}}$ and ${\textbf {b}}$ are defined as:

37 $$\begin{aligned} \begin{aligned} {\textbf {a}} = \left( \begin{array}{c} \sin \theta \, \cos \varphi \\ \sin \theta \, \sin \varphi \\ \cos \theta \end{array}\right) ; \quad 0\le \theta \le \pi , \, 0 \le \varphi \le 2\pi , \end{aligned} \end{aligned}$$

38 $$\begin{aligned} \begin{aligned} {\textbf {b}} = \left( \begin{array}{c} \cos \theta \, \cos \varphi \, \cos \gamma - \sin \theta \, \sin \gamma \\ \cos \theta \, \sin \varphi \, \cos \gamma - \cos \theta \, \sin \gamma \\ -\sin \theta \, \cos \gamma \end{array}\right) ; \quad 0 \le \gamma \le 2 \pi . \end{aligned} \end{aligned}$$

Considering Eq. (36), the spatial dependence of linear compressibility ($\beta$), Young’s modulus (*E*), shear modulus (*G*), and Poisson’s ratio ($\nu$) along the (*xy*), (*xz*), and (*yz*) planes can be defined as follows^94^:

39 $$\begin{aligned} \begin{aligned}&\beta (\theta , \varphi ) = \frac{1}{B} = a_i a_j S_{ijkl}, \end{aligned} \end{aligned}$$

40 $$\begin{aligned} \begin{aligned}&E(\theta , \varphi ) = \frac{1}{S'_{11}(\theta , \varphi )} = \frac{1}{a_i a_j a_k a_l S_{ijkl}}, \end{aligned} \end{aligned}$$

41 $$\begin{aligned} \begin{aligned}&G(\theta , \varphi , \gamma ) = \frac{1}{4 S'_{66}(\theta , \varphi , \gamma )} = \frac{1}{4 a_i b_j a_k b_l S_{ijkl}}, \end{aligned} \end{aligned}$$

42 $$\begin{aligned} \begin{aligned}&\nu (\theta , \varphi , \gamma ) = - \frac{S'_{12}(\theta , \varphi , \gamma )}{S'_{11}(\theta , \varphi )} = - \frac{a_i a_j b_k b_l S_{ijkl}}{a_i a_j a_k a_l S_{ijkl}}, \end{aligned} \end{aligned}$$

where $S_{ij}$ represents the elastic compliance constant, and $l_{1}$, $l_{2}$, and $l_{3}$ are the direction cosines related to the *a*, *b*, and *c* axes of the lattice.

In Fig. 4(a), the spatial dependence of linear compressibility ($\beta$) is depicted. The projection of $\beta$ along the (*xy*), (*xz*), and (*yz*) planes is shown in Figs. 4($\text {a}_{1}$, $\text {a}_{2}$, and $\text {a}_{3}$). These plots reveal that $\beta$ is isotropic in nature, as indicated by the spherical shape of its three-dimensional surface and the circular cross-sections in the 2D projections. Consequently, considering Eq. (39), we can infer that $\text {GdAl}_2$ is also isotropic in terms of its bulk modulus, consistent with previous findings. This isotropy is further supported by Table 5, where $\beta$ exhibits no variation, confirming the isotropic behavior of $\text {GdAl}_2$ in bulk modulus. Quantitatively, $\beta _{\text {min}}=\beta _{\text {max}}=4.131$ $(\text {TPa}^{-1})$ further reinforces the uniform compressibility in all directions. Similarly, the bulk modulus anisotropy index $A_B = 0$ corroborates this uniform compressibility, see Table 4.

The spatial dependence of Young’s modulus (*E*) is shown in Fig. 4(b), with the corresponding (*xy*), (*xz*), and (*yz*) projections displayed in Figs. 4($\text {b}_{1}$, $\text {b}_{2}$, and $\text {b}_{3}$). The slight deviations from circularity suggest minor anisotropy in *E*. Table 5 provides the minimum and maximum values of $\beta$ and *E* for $\text {GdAl}_2$, obtained from the figures. The anisotropy ratio for *E*, computed as $E_{\text {max}}/E_{\text {min}} = 1.057$ ($E_{\text {min}} = 149.57$ $\text {GPa}$, $E_{\text {max}} = 158.11$ $\text {GPa}$), suggests a negligible level of anisotropy, making $\text {GdAl}_2$ nearly isotropic in terms of stiffness. The spatial dependence of the shear modulus (*G*) for $\text {GdAl}_2$ is shown in Fig. 5(a), with the projections along (*xy*), (*xz*), and (*yz*) planes displayed in Figs. 5($\text {a}_{1}$, $\text {a}_{2}$, and $\text {a}_{3}$). These figures indicate that *G* exhibits slight anisotropy, in agreement with the anisotropy index $A_G$ from Table 4. The minimum and maximum values of *G*, as shown in Table 5, suggest that the anisotropy in *G* is minor, with $G_{\text {max}}/G_{\text {min}} = 1.073$ ($G_{\text {min}} = 62.786$ $\text {GPa}$, $G_{\text {max}} = 67.370$ $\text {GPa}$), confirming near-isotropic behavior in terms of shear stiffness. Fig. 5(b) depicts the spatial dependence of Poisson’s ratio ($\nu$), with the corresponding projections shown in Figs. 5($\text {b}_{1}$, $\text {b}_{2}$, and $\text {b}_{3}$). As seen, $\nu$ demonstrates more pronounced anisotropy compared to *E* and *G*. The minimum and maximum values of $\nu$, provided in Table 5, result in an anisotropy ratio $\nu _{\text {max}}/\nu _{\text {min}} = 1.247$ ($\nu _{\text {min}} = 0.166$, $\nu _{\text {max}} = 0.207$), suggesting moderate anisotropy in lateral deformation behavior.

Finally, let us summarize the range of values for linear compressibility ($\beta$), Young’s modulus (*E*), shear modulus (*G*), and Poisson’s ratio ($\nu$) for the $\text {GdAl}_2$ compound, derived from Figs. 4 and 5. The minimum values, represented by the green curves in Figs. 4 and 5, and the maximum values, represented by the blue curves, are provided in Table 5.

### The effects of pressure on the elastic properties

To our knowledge, no prior experimental or theoretical data has investigated the behavior of the elastic properties of $\text {GdAl}_2$ under increasing pressure. Therefore, in this subsection, we analyze the effects of pressure on the elastic properties of this compound, with the aim of providing valuable insights for future studies.

**Fig. 6:**
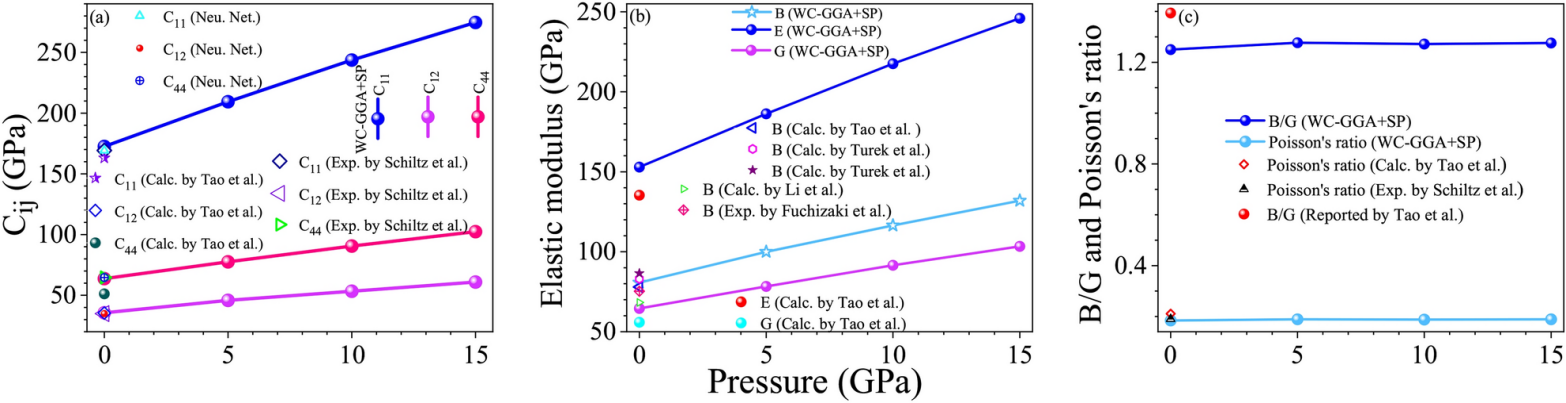
(**a**) Calculated elastic constants, (**b**) elastic moduli, and (**c**) $\frac{B}{G}$ ratio and Poisson’s ratio for $\text {GdAl}_2$ as functions of pressure, determined using the WC-GGA+SP method. Data from other theoretical studies are taken from Refs.^12,17,71^. Experimental data are from Refs.^14,20^. SP denotes spin polarization.

#### Pressure dependence of elastic constants, elastic modulus, B/G, and Poisson’s ratio

Using the WC-GGA method with spin polarization, we examine the pressure dependence of the elastic constants $C_{11}$, $C_{12}$, and $C_{44}$, as shown in Fig. 6(a). Our results reveal a general upward trend for all three constants as pressure increases, indicating that $\text {GdAl}_2$ becomes stiffer under compression. This stiffening effect reflects the strengthening of interatomic interactions under pressure, as increased atomic proximity enhances resistance to deformation. The longitudinal elastic constant $C_{11}$, which represents the material’s response to longitudinal or compressive stress along the primary crystal axis, shows the most significant increase among the three elastic constants with rising pressure. This steep upward trend in $C_{11}$ suggests that $\text {GdAl}_2$ becomes much more resistant to compression along this axis under higher pressure, reflecting a pronounced increase in directional stiffness. Such behavior is characteristic of materials where the primary bonding directions become increasingly resistant to deformation when compressed. This stiffening along the $C_{11}$ direction also implies that $\text {GdAl}_2$ would maintain dimensional stability in applications involving uniaxial compressive forces, as higher pressure enhances the bonding strength and, consequently, the material’s resistance to deformation along the primary axis.

The transverse elastic constant $C_{44}$, which describes the material’s resistance to shear or transverse deformation, increases more gradually with pressure than $C_{11}$. The slower growth rate of $C_{44}$ under compression suggests that $\text {GdAl}_2$ is relatively less responsive to changes in shear deformation resistance compared to longitudinal deformation. Physically, this indicates that while compression strengthens interatomic bonds in the direction of the applied pressure, the atomic layers experience relatively less restriction in their ability to slide past each other under transverse stress. This moderate increase in $C_{44}$ suggests that $\text {GdAl}_2$ retains a certain degree of flexibility in shear deformation, although it becomes progressively more resistant as pressure increases. The off-diagonal elastic constant $C_{12}$, representing the coupling between stress and strain in perpendicular directions, also increases with pressure, though its growth rate lies between that of $C_{11}$ and $C_{44}$. This behavior reflects a moderate increase in resistance to volume-preserving shape changes (such as tetragonal distortions) as pressure rises. The steady increase in $C_{12}$ implies enhanced lateral bonding strength under compression, contributing to the material’s overall stability. However, because $C_{12}$ does not increase as significantly as $C_{11}$, $\text {GdAl}_2$ remains more responsive to shape deformation than purely volumetric compression, highlighting an anisotropic response to pressure.

The increasing values of $C_{11}$, $C_{12}$, and $C_{44}$ under pressure, coupled with their magnitudes satisfying the Born mechanical stability criteria, confirm that $\text {GdAl}_2$ remains mechanically stable up to at least 15 GPa. This stability suggests that the crystal structure can withstand the applied pressures without undergoing a phase transformation or mechanical instability. The lack of sudden changes or anomalies in the trends of $C_{ij}$ further indicates that $\text {GdAl}_2$ retains its crystal structure under compression, making it a viable candidate for applications requiring stability under high pressure. To further reinforce this conclusion, we extended our analysis by recalculating the elastic constants at 20 GPa. Given the computational complexity of high-pressure elasticity calculations, these additional computations provide a more comprehensive assessment of $\text {GdAl}_2$’s mechanical resilience. At 20 GPa, the obtained elastic constants are: $C_{11} = 295.8924 \text { GPa}, \quad C_{12} = 65.0473 \text { GPa}, \quad \text{and} \quad C_{44} = 111.6040 \text { GPa}.$ These values continue the expected increasing trend observed up to 15 GPa, reinforcing that the cubic Laves phase of $\text {GdAl}_2$ remains mechanically robust even under higher compressive stress. The absence of any abrupt anomalies or discontinuities in the pressure-dependent evolution of $C_{ij}$ confirms that $\text {GdAl}_2$ retains its crystal structure and stability across the investigated pressure range. The systematic increase in elastic moduli with pressure indicates a progressive stiffening of the lattice, driven by enhanced interatomic interactions as bond lengths contract under compression. This sustained mechanical robustness is highly relevant for applications requiring stability in environments, including aerospace, deep-sea exploration, and high-performance energy systems. The fact that $\text {GdAl}_2$ remains mechanically stable up to 20 GPa suggests that it can endure substantial mechanical stress without compromising its structural integrity, reinforcing its potential as a high-stability material for extreme conditions.

In fact, Fig. 6(a) shows that as pressure increases, $\text {GdAl}_2$ exhibits enhanced stiffness across all directions, with a particularly strong resistance to longitudinal compression (as shown by $C_{11}$). The gradual increase in $C_{44}$ also suggests that the material, while becoming stiffer, retains a degree of shear flexibility. The steady rise in $C_{12}$ confirms increased stability under pressure in terms of resistance to shape distortions. Together, these trends indicate that $\text {GdAl}_2$ could be suitable for high-pressure applications where longitudinal stability is critical, while still offering some degree of flexibility in shear deformation. In Fig. 6(b), we present the effects of pressure on the bulk modulus (*B*), shear modulus (*G*), and Young’s modulus (*E*) for $\text {GdAl}_2$. The observed increase in all three moduli with pressure confirms that $\text {GdAl}_2$ becomes progressively more resistant to both compression and deformation as pressure rises. This stiffening effect aligns with the behavior of the elastic constants and is consistent with the physical expectation that materials become harder to deform when atomic interactions are intensified under compression.

The Young’s modulus *E*, which quantifies the stiffness of $\text {GdAl}_2$ under uniaxial stress, exhibits the most pronounced increase among the three moduli with rising pressure. This trend suggests that under increasing pressure, $\text {GdAl}_2$ becomes significantly harder to stretch or compress in one direction, indicating an enhanced rigidity in its lattice structure when subjected to uniaxial loading. The steep rise in *E* reflects the material’s increasing resistance to directional deformations, which could be advantageous in applications requiring high structural integrity under directional stresses. The bulk modulus *B*, which represents the material’s resistance to volumetric (compressive) deformation, also increases with pressure but somewhat less steeply than *E*. This behavior implies that while $\text {GdAl}_2$ becomes more resistant to compression, its sensitivity to pressure under hydrostatic conditions is comparatively lower than under uniaxial stress. Given that *B* reflects overall compressive stiffness, its moderate growth with pressure still reinforces $\text {GdAl}_2$’s suitability for applications where resistance to compression is essential, although the effect is not as pronounced as in uniaxial stiffness. The shear modulus *G*, which measures the material’s resistance to shape (or shear) deformation, shows the most gradual increase with pressure among the three moduli. This indicates that while $\text {GdAl}_2$ does become stiffer in response to shear deformation under compression, the effect of pressure on shear rigidity is relatively modest. This lower sensitivity of *G* to pressure suggests that $\text {GdAl}_2$ retains some degree of flexibility in response to shear forces, even as its overall stiffness increases. The fact that *B* and *E* increase more sharply with pressure than *G* suggests that $\text {GdAl}_2$’s resistance to volumetric deformation is more significantly impacted by compression than its resistance to shear deformation. This trend implies that the primary atomic interactions within the crystal structure become more rigid under pressure, particularly in response to isotropic compression, while maintaining a relatively lower sensitivity to directional (shear) forces. This behavior is characteristic of materials with relatively high bulk-to-shear modulus ratios, where the material is better suited to withstand compressive stresses than distortional stresses.

Consequently, Fig. 6(b) demonstrates that as pressure increases, $\text {GdAl}_2$ shows enhanced resistance to both compressive and shape-deforming stresses, with Young’s modulus *E* exhibiting the strongest pressure dependence, followed by the bulk modulus *B*, and then the shear modulus *G*. This hierarchy of pressure sensitivity reflects the material’s significant increase in stiffness under uniaxial stresses, which dominates its overall response to pressure. The comparatively steep rise in *E* underscores the material’s enhanced rigidity against directional deformation, while the moderate growth of *B* highlights its strong but comparatively lower sensitivity to isotropic compression. In contrast, the more gradual increase in *G* suggests that $\text {GdAl}_2$ retains some degree of flexibility in response to shear forces, even as its overall stiffness increases. These characteristics highlight $\text {GdAl}_2$ as a mechanically robust material under high-pressure conditions, particularly where enhanced resistance to uniaxial or volumetric compression is critical, while maintaining modest adaptability to shear deformation.

**Fig. 7:**
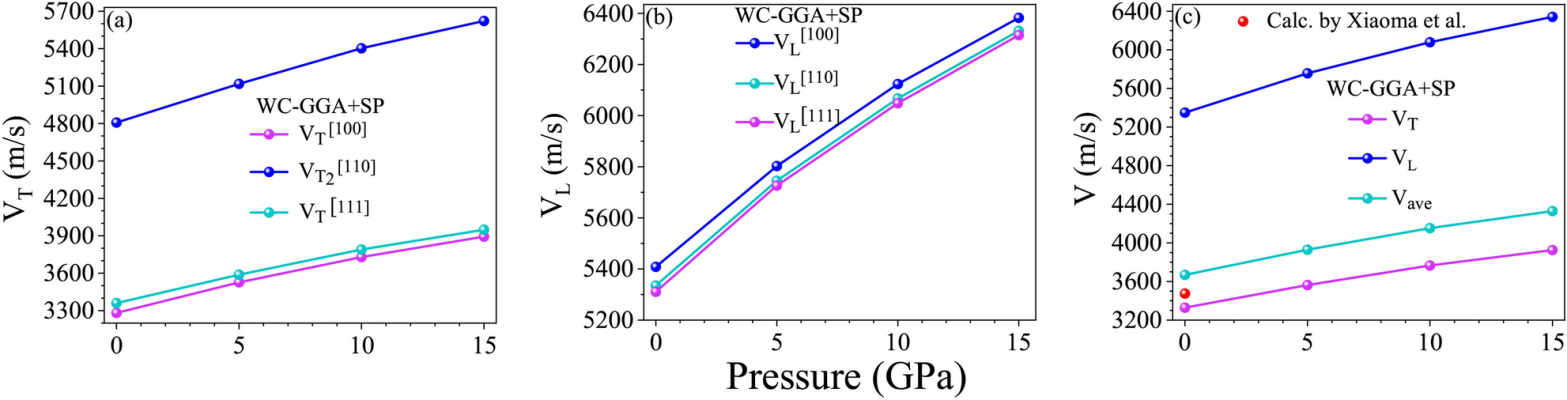
(**a**) Transverse wave velocity ($V_{T}$) calculated using Eq. (29), (**b**) longitudinal wave velocity ($V_{L}$) calculated using Eq. (28), and (**c**) elastic wave velocities in various crystallographic directions using Eq. (20) to Eq. (26), along with the average wave velocity calculated using Eq. (27) for $\text {GdAl}_2$ as functions of pressure, determined with the WC-GGA+SP method. Data for the average wave velocity from other work is taken from Ref.^12^. SP denotes spin polarization.

In Fig. 6(c), we examine the effects of pressure on the ductility indicators for $\text {GdAl}_2$, specifically the *B*/*G* ratio and Poisson’s ratio ($\nu$). Both parameters serve as critical indicators of material ductility versus brittleness, providing insight into the mechanical response of $\text {GdAl}_2$ under increasing pressure. The *B*/*G* ratio is a widely used metric for assessing material ductility; values above 1.75 typically indicate ductile behavior, while values below this threshold suggest brittleness. Across the entire range of pressures studied, the *B*/*G* ratio for $\text {GdAl}_2$ remains consistently below 1.75, strongly indicating that the material retains its inherent brittleness even under compression. This consistent behavior highlights the intrinsic brittleness of $\text {GdAl}_2$, which does not exhibit any transition to a more ductile phase or structure as pressure increases. The low *B*/*G* ratio suggests that the atomic structure and bonding characteristics in $\text {GdAl}_2$ do not accommodate significant plastic deformation, leading to a limited ability to absorb energy without fracturing under stress. Poisson’s ratio ($\nu$) offers another perspective on material ductility. Generally, Poisson’s ratios greater than 0.3 are associated with ductile materials, as they indicate substantial lateral expansion in response to uniaxial compression. In contrast, values below 0.3 are characteristic of brittle materials, where lateral expansion is limited, reflecting a higher tendency to fracture rather than plastically deform. For $\text {GdAl}_2$, Poisson’s ratio remains below 0.3 throughout the studied pressure range, further corroborating its brittle nature. This low Poisson’s ratio suggests that when $\text {GdAl}_2$ is subjected to compressive forces, it exhibits minimal transverse deformation, aligning with the behavior expected from a brittle material.

Thus, the results from Fig. 6 reveal that $\text {GdAl}_2$ exhibits increased stiffness as pressure rises, evidenced by the upward trends in its elastic constants ($C_{11}$, $C_{12}$, and $C_{44}$) and moduli (bulk modulus *B*, shear modulus *G*, and Young’s modulus *E*). This trend indicates a strengthening of atomic interactions within $\text {GdAl}_2$, enhancing its resistance to both compressive and shear deformations. Specifically, $C_{11}$, which is most sensitive to longitudinal compression, shows the largest increase, suggesting a notable enhancement in resistance to uniaxial compression. Conversely, the more gradual increase in $C_{44}$ implies that the material’s response to shear deformation is less influenced by pressure, underscoring a directional dependence in its stiffness characteristics. Despite this stiffening under pressure, $\text {GdAl}_2$ remains inherently brittle, as shown by its consistently low *B*/*G* ratio (below 1.75) and Poisson’s ratio (below 0.3) across all pressures. These values confirm that, while $\text {GdAl}_2$ becomes harder to compress and deform, its fundamental brittleness—rooted in its atomic structure and bonding properties—remains unaffected. The localized nature of Gd’s 4f orbitals likely contributes to this behavior, as these orbitals create rigid bonds that resist deformation but lack flexibility, limiting the material’s capacity for plastic deformation or ductility. The observed anisotropy in wave velocities, with the [110] direction displaying the highest resistance to both shear and compressive deformations, further highlights the directional dependency of $\text {GdAl}_2$’s mechanical properties. Such anisotropy may be advantageous in applications that benefit from selective directional stiffness, especially in high-pressure environments where rigidity is essential. Hence, Fig. 6 provides an analysis of $\text {GdAl}_2$’s pressure-dependent mechanical properties. The material’s increased stiffness under pressure is balanced by a persistent brittleness, making it suitable for applications that require high modulus and rigidity but do not depend on ductility or toughness. For applications needing impact resistance or flexibility, alternative materials or composite modifications would be necessary to overcome the inherent brittleness of $\text {GdAl}_2$.

#### Pressure dependence of sound velocities

In our study, we examined the effect of pressure on phonon wave velocities in various crystallographic directions, focusing on transverse elastic wave velocity ($V_{T}$), longitudinal elastic wave velocity ($V_{L}$), and the average wave velocity ($V_{ave}$) in $\text {GdAl}_2$ up to a pressure of 15 GPa. The results are presented in Fig. 7(a), (b), and (c), respectively.

In Fig. 7(a), we examine the transverse wave velocities $V_{T}^{[100]}$, $V_{T2}^{[110]}$, and $V_{T}^{[111]}$ as functions of pressure for $\text {GdAl}_2$. The results show a clear increase in transverse velocities across all directions with rising pressure, indicating that the material’s resistance to shear deformation strengthens under compression. This trend aligns with the increase in the relevant elastic constants, particularly $C_{44}$, which directly influences the transverse velocity $V_{T}^{[100]}$ (Eq. (20)) and one of the transverse velocities in the [110] direction, $V_{T1}^{[110]}$ (Eq. (22)). Analyzing the individual transverse velocities, we see that $V_{T2}^{[110]}$ consistently exhibits the highest values, followed by $V_{T}^{[111]}$ and $V_{T}^{[100]}$. This hierarchy of velocities highlights anisotropy in the shear response of $\text {GdAl}_2$, with the [110] direction exhibiting the greatest shear stiffness. Specifically, $V_{T2}^{[110]}$ depends on $C_{11} - C_{12}$ (Eq. (23)), suggesting that the combined effect of these constants contributes to a stronger resistance to transverse deformation along the [110] direction compared to [100] and [111]. The elevated values of $V_{T2}^{[110]}$ indicate that atomic bonding along the [110] direction is structurally stiffer or more closely packed, allowing it to better resist transverse deformation under pressure. This anisotropic behavior can be attributed to the unique bonding and atomic arrangements within $\text {GdAl}_2$, which lead to direction-dependent mechanical responses. For the [111] direction, the transverse velocity $V_{T}^{[111]}$ depends on a combination of $C_{11}$, $C_{12}$, and $C_{44}$ (Eq. (25)), reflecting an intermediate resistance to shear deformation due to this complex interplay of elastic constants. Consequently, the differences in $V_{T}$ values for each direction reflect the material’s inherent structural anisotropy, which becomes increasingly pronounced under higher pressures. The increase in transverse velocities $V_{T}$ with pressure further suggests that $\text {GdAl}_2$ could be advantageous in applications that require materials with high resistance to shear deformation, especially in conditions involving directional stress. The observed anisotropy could also inform the design of components that need to withstand shear forces preferentially along specific crystallographic orientations. Therefore, Fig. 7(a) not only confirms that $\text {GdAl}_2$ becomes more shear-resistant with pressure but also underscores the directional dependence of this property-a key consideration for applications in high-pressure environments where mechanical anisotropy might be exploited.

To provide a more detailed interpretation of Fig. 7(b), we analyze the longitudinal wave velocities in $\text {GdAl}_2$ along the [100], [110], and [111] crystallographic directions—denoted as $V_{L}^{[100]}$, $V_{L}^{[110]}$, and $V_{L}^{[111]}$, respectively—as functions of pressure. The consistent increase in $V_{L}$ across all directions with rising pressure indicates that $\text {GdAl}_2$ becomes stiffer under compression, displaying increased resistance to compressive deformation at elevated pressures. Among these directions, $V_{L}^{[100]}$ consistently shows the highest values at each pressure, suggesting that the [100] orientation has the greatest stiffness against compressive forces. This slight directional dependence in the longitudinal wave velocities reflects mild anisotropy in the compressive stiffness of $\text {GdAl}_2$, where atomic interactions along the [100] plane contribute to slightly stronger bonding or more efficient load distribution under compression compared to the [110] and [111] directions. However, it’s important to note that in Fig. 7(a), the transverse velocities exhibit more pronounced anisotropy, with $V_{T}^{[110]}$ being significantly higher than $V_{T}^{[100]}$ and $V_{T}^{[111]}$ at each pressure. This difference suggests that the material’s response to shear stress (transverse deformation) is more directionally dependent than its response to compressive stress. Additionally, the relatively rapid increase in $V_{L}^{[100]}$ with pressure aligns with the marked rise in the $C_{11}$ elastic constant under compression, as $C_{11}$ directly influences stiffness along the [100] axis. This correlation between $V_{L}^{[100]}$ and $C_{11}$ further highlights the role of elastic constants in shaping wave propagation characteristics. The consistent hierarchy in wave velocities–where $V_{L}^{[100]}> V_{L}^{[110]} > V_{L}^{[111]}$–illustrates a structural tendency within $\text {GdAl}_2$ for accommodating compressive stress with the highest resistance along the [100] direction. This combination of mild anisotropy in compressive stiffness with more pronounced anisotropy in shear stiffness may be advantageous in applications that require resistance to directional shear forces while maintaining relatively uniform compressive strength. Overall, the data suggest that $\text {GdAl}_2$ could exhibit varied mechanical responses under applied forces depending on the crystallographic direction, making it suitable for structural or high-pressure applications involving this material.

**Fig. 8:**
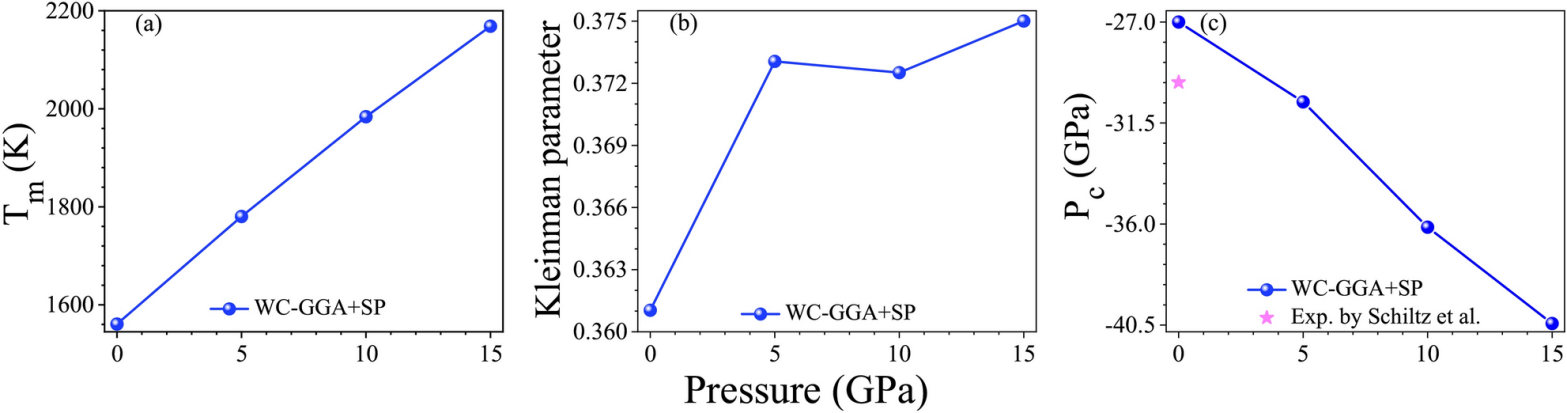
(**a**) Melting temperature ($T_{m}$) calculated using Eq. (30), (**b**) Kleinman’s internal displacement parameter ($\zeta$) calculated using Eq. (18), and (**c**) Cauchy pressure ($P_{c}$) calculated using Eq. (6) for $\text {GdAl}_2$ as functions of pressure, obtained with the WC-GGA+SP method. Experimental data are sourced from Ref.^20^. SP indicates spin polarization.

To provide a more comprehensive analysis of Fig. 7(c), we examine the calculated transverse ($V_T$), longitudinal ($V_L$), and average wave velocities ($V_{ave}$) of $\text {GdAl}_2$ as a function of pressure, along with comparison data from Ref.^12^. As illustrated in the figure, both $V_T$ and $V_L$ increase with pressure, indicating that $\text {GdAl}_2$ exhibits enhanced resistance to both transverse and compressive deformations as pressure rises. Among the three, $V_L$ displays the highest values, followed by $V_{ave}$, with $V_T$ consistently the lowest. This ordering suggests that $\text {GdAl}_2$ is considerably more resistant to compressive wave propagation than to transverse wave propagation, a property that becomes increasingly pronounced with pressure. The comparison with the calculated data from Xiaoma *et al.*^12^ reveals an alignment between their results and our computed $V_{ave}$ values obtained via the WC-GGA+SP method. This agreement with previous theoretical data not only reinforces the reliability of the WC-GGA+SP approach but also validates our computational method for predicting elastic wave velocities under pressure. The consistent match between our calculated $V_{ave}$ values and Xiaoma *et al.*’s data^12^ implies that our approach accurately captures the mechanical response of $\text {GdAl}_2$ to pressure. The upward trend in $V_{ave}$ with pressure is in line with the previously observed increases in elastic constants and moduli, further confirming that $\text {GdAl}_2$ becomes stiffer across all directions as pressure is applied. The consistent increase in wave velocities reflects the overall mechanical robustness of $\text {GdAl}_2$ under pressure, supporting its potential for applications where resistance to compressive forces is critical. However, the pronounced difference between $V_L$ and $V_T$ underscores an inherent anisotropy in the material’s response to stress, indicating that the material’s structural stiffness is more effective in opposing compressive deformation than shear deformation.

The results across all panels in Fig. 7 clearly demonstrate that as pressure increases, $\text {GdAl}_2$ undergoes a pronounced stiffening, evidenced by higher phonon velocities in both transverse and longitudinal directions. This pressure-induced enhancement in wave velocities signifies an increase in resistance to deformation, with the material becoming progressively more rigid under compressive forces. The observed anisotropy in wave propagation, however, varies between the transverse and longitudinal modes. In the case of transverse wave velocities, $V_{T2}^{[110]}$ generally exhibits the highest values among the transverse modes, suggesting that shear resistance is slightly greater along the [110] direction. However, this trend does not apply uniformly across all directions and pressure levels, as the [100] and [111] directions exhibit comparable resistance at lower pressures. For longitudinal wave velocities, $V_{L}^{[100]}$ consistently shows the highest values at each pressure, indicating that the compressive stiffness is greatest along the [100] direction. This variation between transverse and longitudinal behavior highlights a nuanced anisotropy in $\text {GdAl}_2$’s mechanical response, where different directions exhibit unique resistance characteristics depending on the type of deformation. Such selective anisotropic stiffening under pressure could make $\text {GdAl}_2$ particularly suitable for applications where directional mechanical properties are advantageous. In high-pressure environments, this anisotropy could be exploited to enhance rigidity or selective resistance to deformation, depending on the orientation and type of applied stresses. The close agreement of our computed average wave velocities with data from previous studies further reinforces the reliability of our method and supports the potential use of $\text {GdAl}_2$ in applications where stability and anisotropic mechanical performance are critical.

#### Pressure dependence of melting temperature, Kleinman parameter, and Cauchy pressure

In Fig. 8, we analyze the melting temperature ($T_{m}$), Kleinman’s internal displacement parameter ($\zeta$), and Cauchy pressure ($P_{c}$) for $\text {GdAl}_2$ as functions of pressure, calculated using the WC-GGA+SP method. In Fig. 8(a), the melting temperature ($T_{m}$) of $\text {GdAl}_2$ shows a clear linear increase with pressure, starting from approximately 1600 K at 0 GPa and reaching around 2100 K at 15 GPa. This behavior reflects the strengthening of atomic bonds under compression, where higher thermal energy is needed to overcome the increased bonding forces and induce melting. Physically, this linear trend suggests that the material’s lattice becomes more resistant to thermal disruption as pressure rises, reinforcing its structural integrity in high-pressure conditions. The increase in $T_{m}$ indicates that $\text {GdAl}_2$ becomes more thermally stable with pressure, a property beneficial for materials used in high-stress environments. The enhanced melting temperature suggests that $\text {GdAl}_2$ could withstand higher operational temperatures without undergoing phase changes, making it an ideal candidate for applications where both high pressure and high temperature are prevalent. This characteristic highlights $\text {GdAl}_2$’s potential in demanding applications such as aerospace, nuclear reactors, and deep-sea engineering, where materials must withstand high pressures, elevated temperatures, and chemically corrosive or mechanically strenuous environments. Its ability to maintain stability at elevated temperatures and pressures could help improve the longevity and reliability of components in these fields. Additionally, the predictable linear increase in $T_m$ provides designers and engineers with a clear understanding of the material’s performance limits, facilitating its integration into systems where precise temperature tolerance is critical.

In Fig. 8(b), we observe a gradual increase in the Kleinman parameter, $\zeta$, from approximately 0.361 at ambient pressure to about 0.375 at 15 GPa, suggesting that the atomic positions within $\text {GdAl}_2$ change slightly to lattice distortions under higher pressure. Physically, this gradual increase in $\zeta$ indicates slightly change in the internal flexibility, i.e., the crystal structure does’t allow for significant movement in response to volume-conserving strain. This slightly change of rigidity in the atomic arrangement implies that this material shows the minor adjustments in atomic positioning without fracturing under increasing pressure, which could not change its plastic deformation capacity.

In Fig. 8(c), the Cauchy pressure, $P_{c}$, decreases as pressure increases, from approximately -27.00 GPa at ambient pressure to about -40.43 GPa at 15 GPa. Physically, this persistent negative $P_{c}$ value indicates a covalent bonding character within $\text {GdAl}_{2}$, consistent with Pettifor’s criterion^73^. The further decrease in *P* *c* with pressure suggests an increasing covalent contribution to the bonding network, reinforcing lattice stability and resistance to shear deformation under compression. This strengthening of covalent interactions may also contribute to the brittle nature of $\text {GdAl}_2$, as enhanced covalent bonding generally reduces plasticity. Furthermore, this trend corroborates earlier assessments based on the Kleinman parameter, reaffirming that $\text {GdAl}_2$ retains its covalent bonding nature even at elevated pressures, making it a promising candidate for structural applications in high-stress environments where stability against lattice distortions is crucial.

Overall, Fig. 8 provides a comprehensive view of $\text {GdAl}_2$’s behavior under increasing pressure, revealing a complementary balance of thermal, mechanical, and bonding properties that enhance its applicability in demanding environments. The rising melting temperature in Fig. 8(a) reflects $\text {GdAl}_2$’s enhanced thermal stability, ideal for high-temperature conditions. Meanwhile, the behavior of the Kleinman parameter in Fig. 8(b) and Cauchy pressure in Fig. 8(c), retained covalent bonding and resistance to lattice distortions, positioning $\text {GdAl}_2$ as a candidate for structural applications in high-stress, high-pressure environments.

#### Pressure dependence of elastic anisotropy factors

**Fig. 9:**
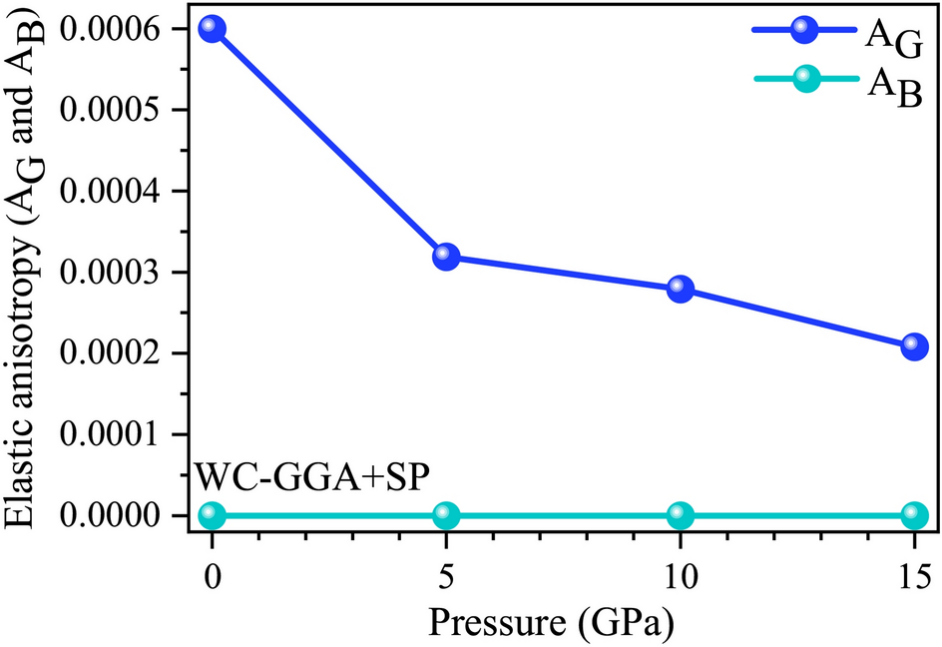
Calculated anisotropy in compression ($A_{B}$) and shear ($A_{G}$) as functions of pressure for $\text {GdAl}_{2}$, using the WC-GGA+SP method based on the two relations expressed in Eq. (34), respectively. $A_{B}$ remains near zero across all pressures, indicating isotropic compressive behavior, while $A_{G}$ exhibits a slight, negligible decrease with increasing pressure, reflecting an almost isotropic shear response. SP denotes spin polarization.

The anisotropy factors $A_{B}$ (compression) and $A_{G}$ (shear) quantify the directional dependence of mechanical properties under pressure. As shown in Fig. 9, $A_{B}$ remains consistently near zero across the entire pressure range, demonstrating the isotropic compressive response of $\text {GdAl}_2$ up to 15 GPa. This indicates that the bulk modulus is nearly uniform in all directions, reflecting a balanced atomic bonding network throughout the crystal. Such isotropy ensures that $\text {GdAl}_2$ maintains its shape and volume uniformly under hydrostatic pressure, a key property for structural applications requiring high volumetric stability in high-pressure environments.

The nearly perfect isotropy in compression ($A_{B} \approx 0$) highlights $\text {GdAl}_2$’s suitability for applications where uniform volumetric stability is critical, such as in high-precision components in aerospace or deep-sea environments. Its consistent performance under hydrostatic pressure minimizes internal stresses and distortion, ensuring durability and reliability in demanding settings. In contrast, $A_{G}$, which starts at a low value (0.0006) at ambient pressure, exhibits a negligible decrease with increasing pressure. This minimal variation implies that $\text {GdAl}_2$ also demonstrates near-isotropic behavior in response to shear stress, with only minor directional differences in resistance to deformation. Such a consistent shear response ensures the material’s structural integrity under complex loading conditions, making it ideal for components exposed to simultaneous compressive and shear forces.

The combined behavior of $A_{B}$ and $A_{G}$ underscores the inherent isotropy of $\text {GdAl}_2$’s mechanical properties under pressure. The material’s ability to maintain uniform compressive and shear resistance across varying directions provides predictable and reliable performance, especially in high-stress environments. This dual isotropy makes $\text {GdAl}_2$ a promising candidate for advanced engineering applications requiring robust and direction-independent mechanical stability, such as structural supports, aerospace components, and pressure-resistant devices.

#### Pressure dependence of phonon dispersion and DOS

**Fig. 10:**
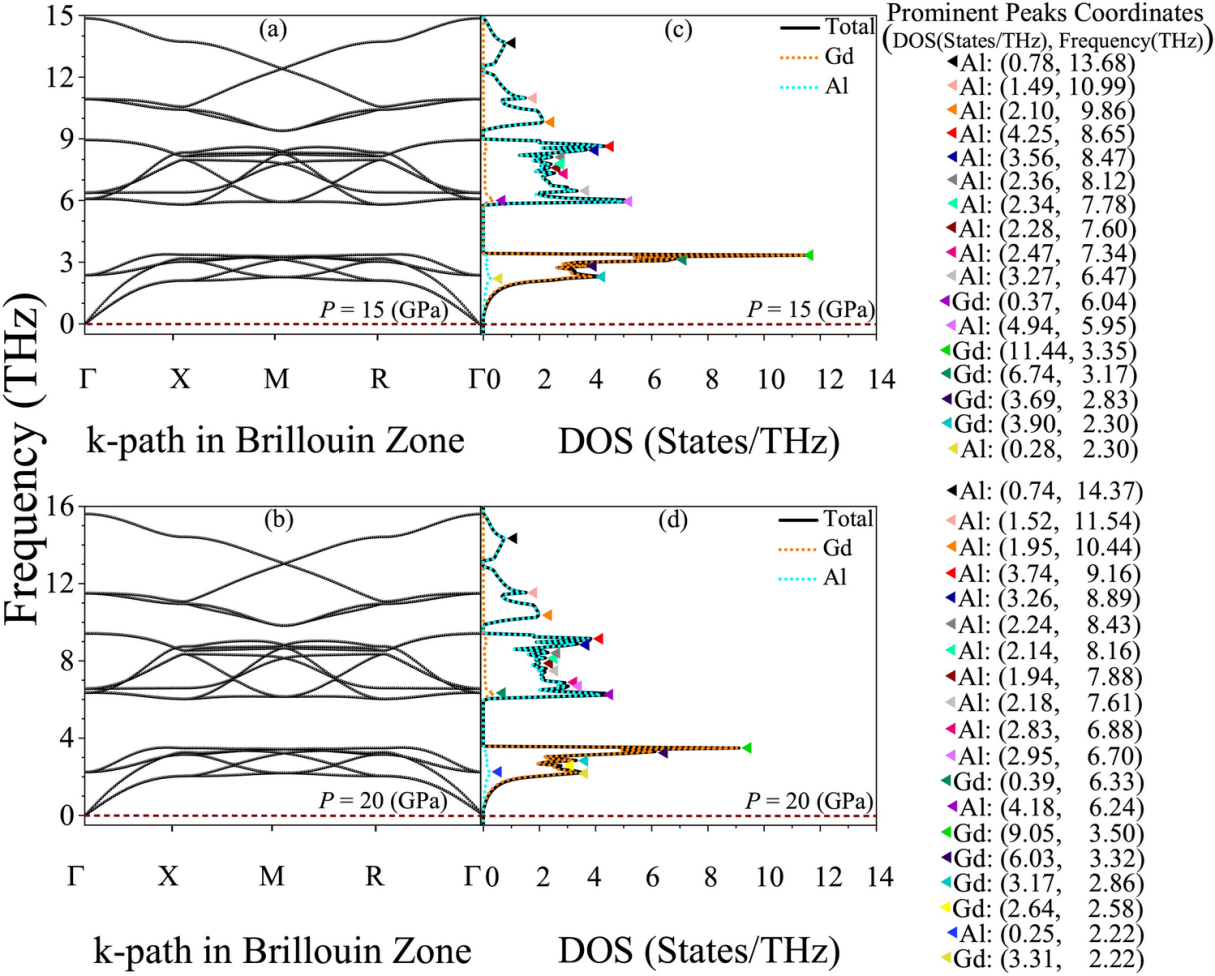
Phonon dispersion curves of $\text {GdAl}_2$ at (**a**) 15 GPa and (**b**) 20 GPa, illustrating the variations of phonon frequency with wave vector across different directions in the Brillouin zone. Atom-resolved phonon densities of states (DOSs) for $\text {GdAl}_2$ at (**c**) 15 GPa and (**d**) 20 GPa, highlighting the contributions of Gd and Al atoms to the phonon spectrum. The prominent peaks and their coordinates in the DOSs-frequency space are explicitly marked in the figure legends. Calculations were performed using the WC-GGA+SP method, where SP denotes spin polarization.

The phonon dispersion and atom-resolved phonon DOS of $\text {GdAl}_2$ at zero pressure are presented in Fig. 3, while the results at two elevated pressures (15 and 20 GPa) are shown in Fig. 10. The results indicate a systematic evolution of vibrational properties with increasing pressure, highlighting significant changes in phonon frequencies, atomic contributions, and mechanical stability. A key observation is the overall blue shift in phonon frequencies with increasing pressure, indicating enhanced interatomic interactions and bond stiffening. The maximum phonon frequency increases from 12 THz at zero pressure, as shown in Fig. 3(a), to 15 THz at 15 GPa, as shown in Fig. 10(a) and further to 16 THz at 20 GPa, as shown in Fig. 10(b), reflecting a progressive hardening of the phonon spectrum. This trend suggests that compression strengthens the atomic bonding, reducing lattice anharmonicity and increasing vibrational energy. The phonon dispersion curves also exhibit steepening of the acoustic branches under pressure, which is directly linked to enhanced elastic constants. At zero pressure, the acoustic phonon modes extend up to approximately 3 THz, as shown in Fig. 3(a), while at 15 GPa and 20 GPa, this range increases to 3.2 and 3.3 THz, as shown in Figs. 10(a) and (b), respectively. The increased slope of acoustic branches under compression implies higher sound velocities, indicating stronger mechanical rigidity and resistance to deformation. The absence of imaginary phonon frequencies across all pressures confirms the dynamical stability of $\text {GdAl}_2$ under compression, ensuring its robustness in high-pressure environments, see Fig. 3(a) and Figs. 10(a) and (b).

The phonon DOS analysis reveals significant pressure-induced shifts in the atom-resolved contributions of Gd and Al atoms. In the low-frequency region (0-4 THz at all pressures), Gd dominates the vibrational spectrum due to its higher atomic mass. The most prominent Gd peak shifts from 3.20 THz (11.69 States/THz) at 0 GPa to 3.35 THz (11.44 States/THz) at 15 GPa and further to 3.50 THz (9.05 States/THz) at 20 GPa, see Fig. 3(b) and Figs. 10(c) and (d). This systematic increase in peak frequency highlights a systematic blue shift with increasing pressure while also showing a reduction in intensity. This shift reflects the stiffening of Gd-related vibrations under compression, contributing to the overall mechanical stability of the structure at elevated pressures. In contrast to Gd, Al contributions in the low-frequency region remain relatively minor. At 0 GPa, a small Al peak is observed at 3.06 THz with a DOS value of 0.46 States/THz, as shown in Fig. 3(b). Under compression, this peak shifts to 2.30 THz (0.28 States/THz) at 15 GPa, Fig. 10(c), and further to 2.22 THz (0.25 States/THz) at 20 GPa, as shown in Fig. 10(d), while consistently remaining significantly weaker than the Gd-related contributions. This trend indicates that Al plays a minimal role in low-energy lattice vibrations, and its influence becomes even less pronounced at higher pressures. Additionally, the systematic decrease in the DOS values from 0.46 States/THz at 0 GPa to 0.28 States/THz at 15 GPa and 0.25 States/THz at 20 GPa further reinforces the observation that Al’s contribution to the vibrational density of states diminishes under compression, see Fig. 3(b) and Figs. 10(c) and (d).

The intermediate frequency range, marking the transition from acoustic to optical modes, expands with increasing pressure, shifting from [4-7 THz] at 0 GPa to [4-9 THz] at 15 GPa and further to [4-9.5 THz] at 20 GPa. Within this region, the contribution of Al to lattice vibrations becomes increasingly significant under compression. At 0 GPa, the most prominent Al peak appears at 5.56 THz with a DOS value of 12.98 States/THz, as shown in Fig. 3(b). With increasing pressure, this peak shifts to 5.95 THz (4.94 States/THz) at 15 GPa and further to 6.24 THz (4.18 States/THz) at 20 GPa, as seen in Figs. 10(c) and (d). This systematic blue shift of Al-related phonon modes indicates that Al atoms play an increasingly dominant role in high-energy vibrational modes under compression. Conversely, Gd contributions in the intermediate frequency range exhibit a contrasting trend. At 0 GPa, a minor Gd peak appears at 5.00 THz with a DOS value of 0.69 States/THz, signifying limited involvement in mid-frequency vibrations. Under compression, this peak shifts slightly to 6.04 THz (0.37 States/THz) at 15 GPa and further to 6.33 THz (0.39 States/THz) at 20 GPa. These values, extracted from Fig. 3(b) and Figs. 10(c) and (d), indicate that while Gd modes also experience a slight blue shift, their overall contribution to the phonon DOS in this frequency range remains relatively weak compared to Al. The decreasing DOS values for Gd in the intermediate region further reinforce the growing dominance of Al in higher-frequency lattice vibrations. This pressure-induced spectral shift highlights the increasing role of Al in the optical phonon spectrum, emphasizing its growing influence in high-frequency lattice vibrations. These trends may have important implications for the mechanical and thermal properties of $\text {GdAl}_2$, particularly in applications where high-pressure stability and vibrational characteristics are critical.

In the high-frequency region, where optical phonon modes dominate, Al atoms play a crucial role due to their lighter mass, which favors high-energy lattice vibrations. This region expands with increasing pressure, shifting from [7-12 THz] at 0 GPa to [9-15 THz] at 15 GPa and further to [9.5-16 THz] at 20 GPa, as seen in Fig. 3(b) and Figs. 10(c) and (d). The most prominent Al peak appears at 7.78 THz (3.89 States/THz) at 0 GPa, shifting to 9.86 THz (2.10 States/THz) at 15 GPa and further to 10.44 THz (1.95 States/THz) at 20 GPa. This systematic blue shift of Al-related optical modes indicates an increase in phonon frequencies under compression, suggesting enhanced interatomic interactions and a rising phonon scattering rate, which could significantly impact thermal transport properties. Conversely, Gd contributions in the high-frequency region remain minimal due to its heavier atomic mass, which limits its participation in high-energy vibrations. At 0 GPa, the DOS of Gd is negligible in the high-frequency region, and this trend persists under pressure, confirming that high-frequency vibrational dynamics are almost exclusively governed by Al atoms. The increasing dominance and strengthening of Al vibrations with pressure further emphasize its crucial role in shaping the high-frequency optical phonon spectrum, which is essential for understanding the mechanical resilience and thermal transport behavior of $\text {GdAl}_2$.

The mechanical stability and elastic properties of $\text {GdAl}_2$ are significantly enhanced under pressure, as evidenced by the steepening of the acoustic branches. The increased phonon frequencies indicate reduced phonon lifetimes, which could lower thermal conductivity at high pressures. The shift in Al-related modes suggests stronger phonon scattering, which can impact thermal transport efficiency, particularly in high-frequency phonon interactions. These results suggest that $\text {GdAl}_2$ exhibits increased stiffness under compression, making it well-suited for high-pressure applications where mechanical resilience is required. The increasing contribution of Al to optical phonons under pressure suggests potential tunability for thermal management applications, where controlling phonon scattering is crucial for optimizing heat conduction. The overall findings indicate that pressure-induced modifications in phonon dispersion and DOS significantly impact the mechanical and thermal properties of $\text {GdAl}_2$. The shift toward higher phonon frequencies confirms enhanced bond stiffness, while the increasing dominance of Al at high frequencies suggests a greater role in phonon scattering processes. The material remains dynamically stable up to at least 20 GPa, making it a viable candidate for applications in extreme environments, including high-pressure structural materials, thermal management systems, and phononic devices requiring tunable vibrational properties. These results provide a roadmap for designing pressure-tuned materials with tailored phonon interactions, where a careful balance of acoustic and optical phonon contributions can be leveraged to optimize mechanical strength and thermal transport behavior.

The possibility of a phase transition in $\text {GdAl}_2$ at pressures beyond 20 GPa remains an open question. However, within the pressure range investigated in this study (0-20 GPa), our phonon calculations confirm the dynamical stability of the cubic Laves phase. Further exploration of high-pressure phase behavior is an interesting direction for future research.

### Temperature dependence of elastic constants and Cauchy pressure

As highlighted in the abstract, $\text {GdAl}_2$ is a promising material for aviation and military applications, particularly in high-pressure environments up to 15 GPa. While pressure-dependent elastic behavior has been thoroughly addressed in Sec. 3.8, assessing the temperature dependence of its mechanical and thermal properties is equally vital for evaluating its suitability in real-world operational scenarios. Standard DFT calculations, though informative under ideal conditions, are typically performed at zero temperature and lack the ability to capture temperature-induced property variations essential for high-performance applications. To bridge this gap, we have referenced methodologies for incorporating temperature effects into DFT calculations in Sec. II.1 of the Electronic Supplementary Information in Ref.^96^, and have built upon our prior work analyzing temperature-dependent properties across various systems^97–103^, as well as recent investigations of magnetization behavior in LiTmF$_4$^104^. Motivated by these studies and our recent experience^30^, we adopt a machine-learning-based strategy using a feedforward multi-layer perceptron neural network, described in Sec. 2.2, to model the temperature dependence of the elastic constants of $\text {GdAl}_2$. Neural networks are particularly well-suited for capturing complex nonlinear relationships. In this study, our model was trained on reliable experimental data from Ref.^20^ and implemented within a Bayesian regularization framework to mitigate overfitting and enhance generalization. Implemented in PyTorch^105,106^, the architecture comprises two hidden layers with 16 ReLU-activated neurons each and an output layer for $C_{11}$, $C_{12}$, and $C_{44}$. The total loss function, $\text {Total Loss} = E_D + \lambda E_W$, balances data fidelity and model simplicity by minimizing prediction error and penalizing large weights. This strategy outperforms traditional methods by combining experimental reliability and machine learning’s flexibility for interpolation and extrapolation. The resulting predictions were further fitted to analytical forms, including second- and third-order polynomials and two empirical models proposed by Varshni^95^, which have proven effective across multiple materials:

43 $$\begin{aligned} \begin{aligned}&C_{ij}(T) = C_{ij}^0 - \frac{s}{e^{t/T} - 1}, \end{aligned} \end{aligned}$$

44 $$\begin{aligned} \begin{aligned}&C_{ij}(T) = a - \frac{bT^2}{T + c}, \end{aligned} \end{aligned}$$

where $C_{ij}^0$, *s*, *t*, *a*, *b*, and *c* are material-specific constants determined empirically. Here, $C_{ij}^0$ and *a* represent the values of the elastic constants at zero temperature.

**Fig. 11:**
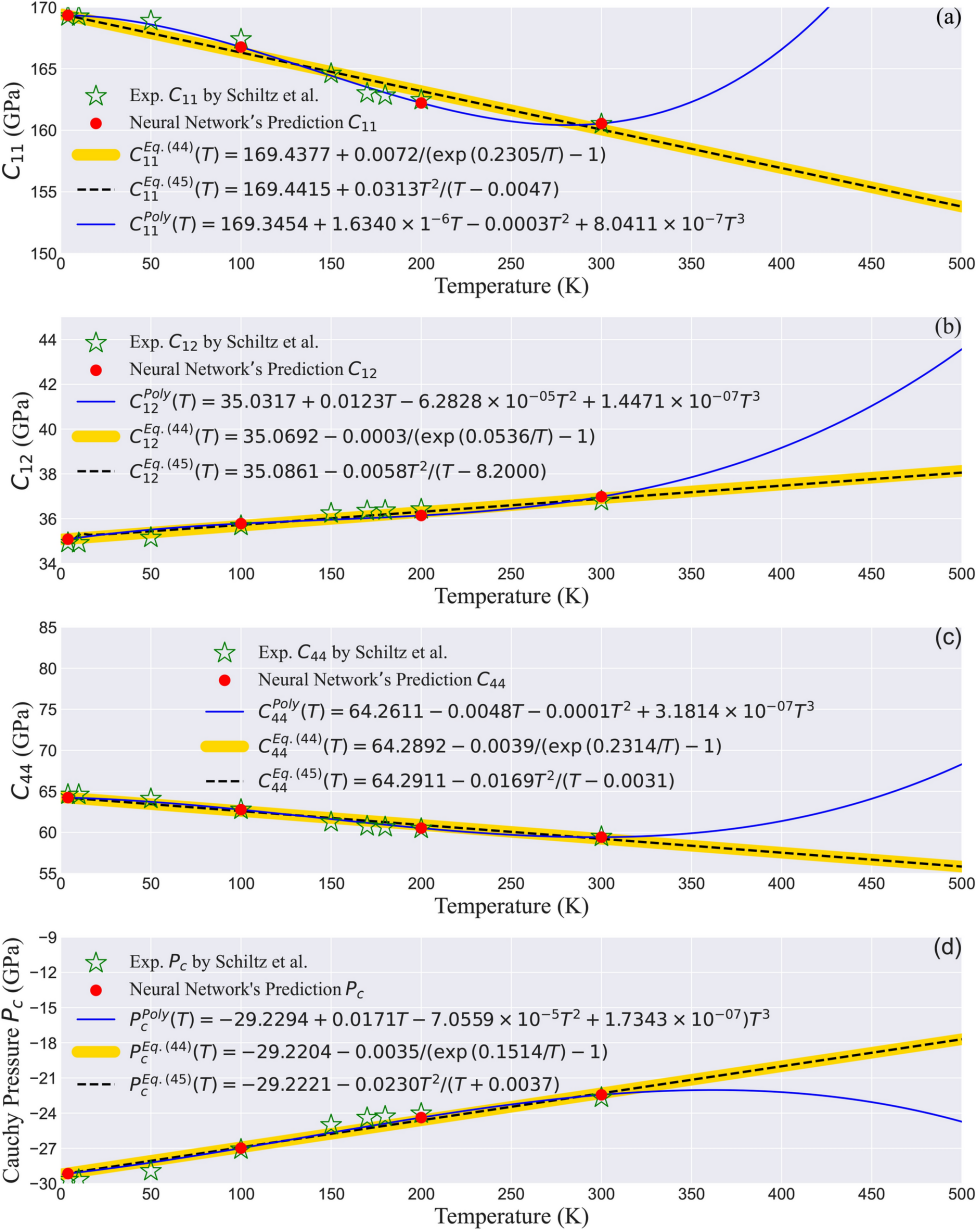
Temperature dependence of the elastic constants: (**a**) $C_{11}$, (**b**) $C_{12}$, (**c**) $C_{44}$, and (**d**) Cauchy pressure ($P_{c}$) for $\hbox {GdAl}_2$. The predictions from the neural network (red filled circles), trained on experimental data (green empty stars) measured by Schiltz et al.^20^, are fitted to three models: the nonlinear rational-exponential equation (Eq. (43), gold solid lines), the nonlinear rational-quadratic equation (Eq. (44), black dashed lines), both proposed by Varshni^95^, and a cubic polynomial fit (blue solid curves).

Fitting the neural network’s predictions to empirical models provides analytical expressions for the temperature dependence of elastic constants. While it may seem reasonable to directly fit experimental data, using a neural network offers distinct advantages: trained on experimental inputs, it captures underlying patterns, including subtle nonlinearities that may not be apparent due to noise or resolution limits. By fitting the network’s outputs, we obtain smooth, robust expressions that generalize well while avoiding the variability of raw data. This strategy also allows meaningful comparison between the neural network predictions and empirical models. Although empirical models offer simplified baselines, they often fall short in representing intricate temperature dependencies, particularly at intermediate temperatures, where neural networks excel. Our neural network results demonstrated excellent agreement with experimental data, with errors below 1% across the full temperature range.

Having established the significance of temperature-dependent elasticity, we now present an in-depth analysis of the elastic constants and Cauchy pressure of $\text {GdAl}_2$, illustrated in Fig. 11. These results yield insights into thermomechanical behavior, mechanical stability, and bonding character under thermal stress. Neural network predictions, trained on experimental data, closely follow empirical trends, validating the method’s reliability. The observed behavior shows that $C_{11}$ and $C_{44}$ systematically decrease with increasing temperature, consistent with weakening interatomic forces due to anharmonic lattice vibrations, while $C_{12}$ exhibits a monotonic increase, suggesting temperature-enhanced shear stiffness for volume-preserving deformations. This contrast highlights a complex interplay of thermal expansion and interatomic interactions. A central feature of this analysis is the fitting of neural network-predicted elastic constants to two empirical models-Eq. (43) and Eq. (44)-both proposed by Varshni^95^. These models are widely adopted for their physical grounding and effectiveness in describing elastic behavior across temperatures. Fitting was performed on neural network outputs rather than directly on experimental data to mitigate noise and extrapolation inconsistencies. This ensures smooth, analytically tractable functions for theoretical modeling and materials design. Specifically, Fig. 11(a) shows $C_{11}$ decreases gradually up to 300 K and then softens more markedly; both empirical models match well, with the rational-quadratic form slightly outperforming at high temperatures. Figure 11(b) highlights the unusual increase of $C_{12}$ with temperature, well captured by both empirical fits, reinforcing the validity of the neural network predictions. In Fig. 11(c), $C_{44}$ displays gradual softening, accurately modeled by the rational-exponential equation, whereas the polynomial fit diverges at elevated temperatures, emphasizing the limitations of polynomials for extrapolation. Furthermore, Fig. 11(d) shows Cauchy pressure $P_c$ remains negative throughout the temperature range, indicating dominant covalent bonding in $\text {GdAl}_2$. The rational-quadratic model better captures subtle trends compared to the polynomial fit, supporting the case for using physically motivated functions. Although similar temperature-dependent analyses could be extended to other elastic properties, we refrain for conciseness and to encourage future exploration. Importantly, polynomial fits-while useful for interpolation-often exhibit unphysical behavior in extrapolation. In contrast, empirical models retain physical validity across broader temperature ranges, strengthening their utility in predictive modeling.

These insights tie back to the broader motivation introduced earlier: understanding temperature-dependent elasticity is essential for evaluating material performance in real-world conditions. Combining neural networks and empirical models bridges the gap between idealized zero-temperature DFT predictions and sparse or noisy experimental data, providing a practical, high-fidelity framework. Additionally, the calculated elastic constants satisfy the Born mechanical stability conditions for cubic crystals^26^, confirming mechanical robustness across all studied temperatures. Collectively, these findings underscore the effectiveness of machine learning in capturing thermomechanical trends and validate the suitability of $\text {GdAl}_2$ for demanding aerospace, defense, and high-temperature engineering applications.

## Multifaceted potential of gadolinium aluminide (GdAl_2_) in advanced engineering and quantum technologies

As we advance toward a future shaped by cutting-edge technologies, materials like gadolinium aluminide ($\text {GdAl}_2$) bridge the gap between theoretical innovation and practical application. Its exceptional combination of mechanical robustness, thermal stability, and magnetic properties positions it as a cornerstone for breakthroughs in quantum computing, high-performance engineering, and sustainable energy systems. This section expands on how these findings translate into practical engineering solutions across a wide spectrum of advanced applications. Gadolinium aluminide exhibits a unique blend of mechanical strength, thermal stability, and magnetic behavior, making it suitable for use in extreme environments and emerging technologies^107,108^. In spintronics, where electron spin is exploited for information processing, the magnetic properties of $\text {GdAl}_2$ provide significant advantages in energy efficiency and processing speed^107^. Additionally, its near-isotropic behavior under varying pressures makes it promising for maintaining qubit coherence in quantum computing, a prerequisite for error correction and operational reliability^107,109,110^.

Mechanically, the low anisotropy and consistent performance of intermetallic compounds like CeAl$_2$ under pressure suggest that $\text {GdAl}_2$ could offer similar resilience, making it ideal for aerospace and deep-sea exploration applications^111^. In thermal management, the distinct vibrational modes of Gd and Al atoms across frequencies enhance phonon control, enabling usage in advanced heat sinks and thermal interface materials for high-performance electronics. The absence of imaginary phonon frequencies in its phonon spectrum confirms its dynamical stability, supporting applications in nuclear reactors and chemical environments requiring resistance to radiation, heat, and corrosion^112^. Its robustness in such extreme environments extends to components like heat shields, turbine blades, and underwater robotics, where it must withstand mechanical stress, high temperatures, and corrosive exposure.

In summary, the versatility of $\text {GdAl}_2$ lies in its ability to meet the multifaceted demands of spintronics, quantum computing, thermal regulation, and structural integrity under extreme thermal and mechanical loads. As research advances, the optimization and integration of its unique properties are expected to broaden its technological impact across several high-performance applications.

## Conclusion

This study unveils the multifaceted potential of the cubic ferromagnetic Laves phase $\text {GdAl}_2$, positioning it as a benchmark material for applications requiring exceptional thermal stability and mechanical robustness in demanding environments. By employing advanced computational methodologies, including density functional theory with multiple exchange-correlation functionals and neural network-based machine learning, we present a comprehensive mechanical profile that paves the way for its deployment in cutting-edge technologies.

Building on our prior research on intermetallic compounds, this work delves deeper into $\text {GdAl}_2$, revealing its unique mechanical profile characterized by near-isotropic compressive resistance and spin-dominated mechanical responses. The balanced isotropy in compressive behavior, coupled with mild anisotropy in shear and Young’s moduli, underscores $\text {GdAl}_2$’s structural and mechanical diversity within the $\text {AB}_2$ family, offering new insights into how symmetry and bonding govern elastic properties.

To address long-standing gaps, we adopted a rigorous energy-based methodology to resolve discrepancies between theoretical and experimental elastic constants, setting a benchmark for cubic systems. Additionally, by investigating the previously unexplored pressure-dependent properties, we established $\text {GdAl}_2$’s mechanical stability under compression up to 15 GPa, revealing consistent increases in elastic constants, bulk modulus, Young’s modulus, shear modulus, and melting temperature, highlighting its resilience in high-stress, high-pressure environments. Extending our analysis further, we also evaluated the mechanical stability at 20 GPa, confirming that $\text {GdAl}_2$ remains robust under even higher pressures, reinforcing its reliability for extreme operational conditions.

Moreover, by incorporating deep learning techniques, we have systematically investigated the temperature dependence of elastic properties, revealing that while $C_{11}$ and $C_{44}$ soften with increasing temperature due to anharmonic lattice vibrations, $C_{12}$ exhibits a monotonic increase, indicating a complex interplay between thermal expansion and shear deformation. The persistence of negative Cauchy pressure confirms that $\text {GdAl}_2$ maintains its dominant covalent bonding character across a broad temperature range, reinforcing its mechanical resilience in thermal conditions. These findings extend the material’s applicability to high-temperature aerospace, nuclear, and geothermal environments where mechanical integrity must be preserved under fluctuating thermal loads.

Key findings include $\text {GdAl}_2$’s exceptional thermal stability, with melting temperatures rising linearly under pressure, and its near-isotropic compressive behavior, coupled with mild anisotropy. These properties make it a strong candidate for aerospace, automotive, geothermal, and other advanced engineering applications requiring materials that endure extreme thermal and mechanical conditions.

Phonon dispersion and density of states analyses further demonstrate that low-frequency acoustic modes dominated by Gd atoms drive elastic behavior, highlighting the material’s spin-dominated mechanics. This simplifies computational modeling while broadening $\text {GdAl}_2$’s applicability to environments requiring both magnetic performance and mechanical stability. Neural network-based predictions validate the computational framework, emphasizing machine learning’s role in accelerating material discovery and design.

While $\text {GdAl}_2$ exhibits remarkable mechanical and thermal properties, its intrinsic brittleness, characterized by a low *B*/*G* ratio and Poisson’s ratio, suggests limited ductility. This brittleness arises from its dominantly covalent bonding nature, as indicated by the negative Cauchy pressure and Kleinman parameter analysis. The strong directional bonding associated with covalent interactions enhances mechanical stability under compression but also reduces plasticity, making $\text {GdAl}_2$ more resistant to dislocation motion and deformation. However, this characteristic does not compromise its pressure stability, as its elastic constants satisfy the Born criteria across all investigated pressures, including 20 GPa. Engineering strategies, such as alloying, nanostructuring, or composite reinforcement, may help mitigate its brittleness, enhancing fracture resistance and toughness for applications requiring high-stress tolerance under impact or dynamic loading.

In summary, this study provides a robust framework for understanding $\text {GdAl}_2$’s unique properties and potential applications. By bridging theoretical and experimental insights, it positions $\text {GdAl}_2$ as a promising material for next-generation aerospace, defense, and energy systems, while paving the way for innovative applications in demanding operational environments.

## Data Availability

All relevant data are provided within the manuscript. Additional information can be made available upon reasonable request by contacting the corresponding author, Prof. Saeid Jalali-Asadabadi, at sjalali@sci.ui.ac.ir or saeid.jalali.asadabadi@gmail.com.
